# Spatiotemporal differential regulation of extrasynaptic GluN2B receptor subunits and PSA-NCAM in brain aging and Alzheimer’s disease

**DOI:** 10.3389/fnins.2025.1649625

**Published:** 2025-08-29

**Authors:** Oghenetega E. Imiruaye, Isis G. Perez, Brian C. Carson, Christian Crouzet, Jerome Garcia, Derick Han, Subhrajit Bhattacharya

**Affiliations:** ^1^Henry E. Riggs School of Applied Life Sciences, Keck Graduate Institute, Claremont, CA, United States; ^2^Beckman Laser Institute and Medical Clinic, University of California, Irvine, Irvine, CA, United States; ^3^College of Arts and Sciences, University of La Verne, La Verne, CA, United States; ^4^School of Pharmacy, Keck Graduate Institute, Claremont, CA, United States

**Keywords:** Alzheimer’s disease, excitatory/inhibitory balance, PSA-NCAM, extrasynaptic GluN2B, brain aging (normal), amyloid–beta, synaptic dysfuction

## Abstract

**Introduction:**

Extrasynaptic GluN2B N-methyl-D-aspartate receptors (ES-GluN2B) are localized outside synapses and promote excitotoxic signaling, apoptosis, and long-term depression (LTD) in Alzheimer’s disease (AD). Polysialylated neural cell adhesion molecule (PSA-NCAM) physiologically inhibits ES-GluN2B activity, and its downregulation is associated with impaired synaptic plasticity. However, the spatiotemporal changes of ES-GluN2B and PSA-NCAM during brain aging versus AD remain poorly understood.

**Methods:**

We investigated GluN2A, GluN2B, ES-GluN2B, and PSA-NCAM expression across brain regions in young and old Tg2576 AD and wild-type (WT) mice. Additional experiments included PSD-95 pulldown assays, analysis of GluN2B phosphorylation at Ser1480, CRISPRa-driven ST8Sia4 upregulation in IMR-32 neuroblastoma cells, and Aβ treatment to assess effects on PSA-NCAM biosynthetic enzymes.

**Results:**

Normal aging was associated with decreased GluN2B, increased GluN2A, stable ES-GluN2B, and elevated PSA-NCAM levels. In contrast, AD aging showed elevated ES-GluN2B and reduced PSA-NCAM, particularly in the hippocampus and cortex, with no change in total NCAM expression. PSD-95 pulldown revealed increased extrasynaptic GluN2B in aged AD brains. AD aging was associated with elevated phosphorylation of GluN2B at Ser1480 by casein kinase 2 (CK2), promoting GluN2B redistribution to extrasynaptic sites. CRISPRa-driven ST8Sia4 upregulation increased PSA-NCAM and reduced pGluN2B expression supporting a direct regulatory role for PSA-NCAM in GluN2B trafficking. Additionally, Aβ suppressed PSA-NCAM biosynthetic enzymes ST8Sia4 and UDP-E linking Aβ to impaired polysialylation.

**Discussion:**

These findings highlight distinct regulatory patterns of ES-GluN2B and PSA-NCAM in AD versus normal aging and support a model in which impaired PSA-NCAM buffering facilitates pathological ES-GluN2B signaling and plasticity loss in AD progression.

## Graphical abstract

A model of differential regulation of ES-GluN2B and modulation of PSA-NCAM in normal aging and AD. *Up*: In normal aging, PSA-NCAM expression increases with aging, while ES-GluN2B expression levels remain unchanged across all brain regions of WT mice. Under these conditions, synaptic plasticity and normal brain function are promoted. *Down*: In AD pathophysiology, ES-GluN2B expression is upregulated, while PSA-NCAM levels are significantly reduced in the investigated brain regions of AD mice. In neuroblastoma cells (IMR-32), Aβ treatment downregulated PSA-NCAM biosynthetic enzymes, ST8Sia4 and UDP-E, to downregulate PSA-NCAM expression.
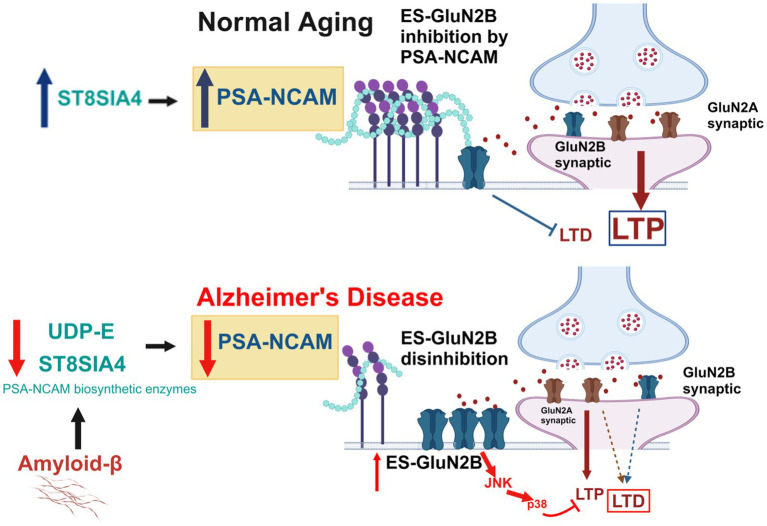

## Highlights

- Altered GluN2A/2B expression and downregulation of GABA_A_R in AD.
- Distinct age-dependent changes in PSA-NCAM and ES-GluN2B in AD and normal aging.
- Enriched ES-GluN2B and downregulated PSA-NCAM expression with AD progression.
- Aβ treatment downregulated ST8Sia4 and UDP-E, to downregulate PSA-NCAM in neuroblastoma cells.

## 1 Introduction

Alzheimer’s disease (AD) is a progressive neurodegenerative disorder marked by memory loss and neuronal dysfunction, strongly associated with aging (Takeda et al., 2008; Rajan et al., 2021). A key feature of AD pathology is the disruption of excitatory-inhibitory (E/I) signaling balance, leading to excitotoxicity and synaptic degeneration (Marín, 2012; Xu et al., 2020). N-methyl-D-aspartate receptors (NMDARs), composed of GluN1 and GluN2 (A-D) subunits (Dingledine et al., 1999), mediate calcium-dependent excitatory transmission and are essential for synaptic plasticity (Bhattacharya et al., 2018a; Bhattacharya et al., 2018b; Traynelis et al., 2020). While GluN2A subunits support neuroprotection and LTP during normal aging, altered GluN2A/B expression and function contribute to AD-related excitotoxicity (Paoletti et al., 2013; Wang and Reddy, 2017). These subunits are differentially expressed across the hippocampus, cortex, and midbrain and vary with age (Monyer et al., 1994; Iwasato et al., 1997; Woodhall et al., 2001), but their regional dynamics in normal vs. AD aging remain underexplored (Li et al., 2011; Ding et al., 2014). In particular, GluN2B-containing NMDARs are distributed both synaptically and extrasynaptically (ES), where tonic, low-level glutamate activation at ES sites primes pro-death signaling pathways including LTD and apoptosis (Hardingham and Bading, 2010; Talantova et al., 2013; Liu et al., 2019a). Phosphorylation of GluN2B at Ser1480 by casein kinase 2 (CK2) drives its extrasynaptic localization, linking this process to the pathogenesis of AD (Sanz-Clemente et al., 2010; Chiu A. M. et al., 2019).

An emerging area of research explores the interaction between polysialylated neural cell adhesion molecule (PSA-NCAM) and extrasynaptic GluN2B (ES-GluN2B) subunits. NCAM is a transmembrane glycoprotein essential for cell adhesion, signaling, and synaptic plasticity (Varbanov and Dityatev, 2017; Giacobbo et al., 2019). Its polysialylation, mediated byST8-*α*-N-acetyl-neuraminide-α-2,8-sialyltransferase-2/4 (ST8Sia2/4) and UDP-N-acetylglucosamine-2-epimerase (UDP-E), modifies NCAM into PSA-NCAM, a key modulator of neurite outgrowth and synaptic remodeling (Giacobbo et al., 2019; Mony and Paoletti, 2023). These changes are critical for synaptogenesis, learning, and memory (Kochlamazashvili et al., 2010; Mota et al., 2014; Chen et al., 2019). ST8Sia2 expression dominates embryonic development, particularly in the hippocampus and cortex, while ST8Sia4 is upregulated postnatally and supports PSA-NCAM expression into adulthood (Murray et al., 2016; Fekete and Nishiyama, 2022). In AD, a reduction in ST8Sia4 mRNA expression has been observed in the entorhinal cortex using multiplex *in situ* hybridization (Highet et al., 2023), complementing prior evidence of decreased PSA-NCAM protein levels in the same region (Murray et al., 2016), suggesting both transcriptional and post-translational disruptions of this regulatory axis. Functionally, PSA-NCAM inhibits ES-GluN2B-driven long-term depression (LTD) and excitotoxic signaling, helping maintain synaptic balance (Dityatev et al., 2004; Senkov et al., 2006; Varbanov et al., 2023). Its loss has been linked to synaptic dysfunction and cognitive decline in aging and AD (Seki and Rutishauser, 1998; Bisaz et al., 2011). Notably, PSA-NCAM can inhibit Ras-p38 MAPK signaling via ES-GluN2Bs, a pro-apoptotic pathway implicated in LTD and cell death (Kochlamazashvili et al., 2010; Varbanov et al., 2023). However, the regional and temporal dynamics of PSA-NCAM and ES-GluN2B interactions across AD-affected brain regions remain poorly defined, warranting further investigation. In this study, we demonstrate the precise spatiotemporal expression patterns of GluN2A/GluN2B-containing NMDARs, ES-GluN2Bs, and PSA-NCAM across brain regions, driving the differential regulation of synaptic and extrasynaptic GluN2B subunits in transgenic and wild-type mouse models. Importantly, we also establish a mechanistic link between PSA-NCAM levels and GluN2B phosphorylation and trafficking, using both *in vivo* (AD and WT mouse brains) and *in vitro* (CRISPRa-ST8Sia4 overexpression) models to show that PSA-NCAM modulation directly regulates Ser1480 GluN2B phosphorylation and ES-GluN2B dynamics. Additionally, using an AD in-vitro model, we demonstrate how amyloid-*β* (Aβ) exposure alters PSA-NCAM levels by downregulating key biosynthetic enzymes ST8Sia4 and UDP-E. Understanding these molecular disruptions clarifies critical differences between normal and AD-related aging and highlights novel therapeutic targets for restoring synaptic plasticity in neurodegeneration.

## 2 Methods

### 2.1 Animals

Male Tg2576 single transgenic AD mice and age-matched wild-type controls (C57BL6; SJL) were obtained from the University of California, Irvine, where they were bred and housed. The study included young (6 months) and aged (16–17 months) cohorts. The *Tg*2576 mice overexpress the human APP gene with the Swedish mutation (KM670/671NL) under the hamster prion promoter (PrP), exhibiting amyloid plaque formation from 11 to 13 months of age and microglial activation in or around plaques in all regions of the cortex and hippocampus from 10 to 16 months of age. The mice received care according to methods approved under institutional guidelines for the care and use of laboratory animals in research. All procedures were carried out in strict accordance with approval by the Institutional Animal Care and Use Committees (IACUC) at the University of California, Irvine. Mice had access to food and water *ad libitum*.

### 2.2 Brain dissections and tissue preparation

Mice were euthanized according to approved protocols, ensuring minimal distress using a CO_2_ chamber. Brains were then dissected to isolate specific brain regions, including the hippocampus (hippo), prefrontal cortex (pfc), cortex (ctx), midbrain (mb), and cerebellum (cbm; as a negative control) as described before (Riga et al., 2014). The brains were gently removed and submerged in ice-cold PBS (in mM: 80.6 NaH_2_PO_4_, 19.4 KH_2_PO_4_, 27 KCl, and 1.37 NaCl, pH 7.4) to minimize tissue degradation. Brains were oriented to identify anatomical landmarks (Riga et al., 2014). Dissected brain regions were immediately placed in separate 1.5 mL microcentrifuge tubes and stored at −80°C. All dissections were performed on ice to maintain tissue integrity, and care was taken to avoid cross-contamination between different brain regions. Following dissection and storage at −80°C, the different brain regions were weighed, and equal volume of lysis buffer containing N-PER™ Neuronal Protein Extraction Reagent (Thermo Scientific, Waltham, MA) and protease/phosphatase inhibitor cocktail (Cell Signaling Technology Inc., MA) was added across all samples in a 1:1 (mass: volume) ratio. The tissue and lysis buffer mixtures were homogenized using an ultrasonic processor (CP-130 PB-1, Cole Parmer, Vernon Hills, IL) set to 20 kHz with an amplitude of 40%. Sonication was performed on ice to prevent overheating, with a pulse on/off cycle of 5 s on and 10 s off, for a total of 2 min. The tissue lysate was briefly spun down to carefully collect the content and stored at −80°C until further analysis.

### 2.3 Immunoblotting – animal tissue

Equal amounts of protein (60 μg) from tissue lysate were loaded for each sample, quantified by Bradford Assay Reagent (Bio-Rad, Hercules, CA), diluted with 5X sample-reducing buffer (Thermo Scientific, Waltham, MA), and normalized to *β*-actin as a loading control. Samples were then either stored at −80 or −20°C for later use or heated at 90°C for 5 min and resolved on 4–20% Bio-Rad Mini-PROTEAN TGX Precast Protein Gradient Gels (Bio-Rad, Hercules, CA) with Precision Plus Protein Dual Color Standard used as a ladder for molecular weight estimation (#1610374, Bio-Rad, Hercules, CA). Samples were run at 70 V for 20 min, 75 V for 20 min, and then at a constant voltage of 80 V until the bands reached the bottom of the gel. Proteins were then transferred to PVDF membranes (0.45-mM pore size, Immuno-Blot, Bio-Rad, Hercules, CA) for Western blotting for 1 h at 100 V. Membranes were blocked in TBS (20 mM Tris, 500 mM NaCl, pH 7.4) containing 1% casein (Bio-Rad, Hercules, CA) and incubated in primary antibodies overnight at 4°C (Table 1). HRP-conjugated goat anti-rabbit secondary (1:2500, Cell Signaling Technology Inc., MA), HRP-conjugated horse anti-mouse secondary (1:2500, Cell Signaling Technology Inc., MA), and HRP-conjugated goat anti-Mouse IgM secondary (1:2500, Thermo Scientific, Waltham, MA) were incubated for 1 h at room temperature. To reevaluate the same membrane, they were exposed to Restore Stripping Buffer (Pierce, 21,059; Thermo Scientific, Waltham, MA) for 10 min, washed with 1xTBS with 1% Tween 20 (TBST), and blocked with 1x TBS with 1% casein again before being re-blotted with the next primary antibodies. Signals from antibodies were imaged with raw films, using an Azure Biosystems C500 Imager (Azure Biosystems, Dublin, CA, United States). The blots were analyzed using Image J software (National Institutes of Health, ImageJ bundled with 64-bit Java 1.8.0_172).

**Table 1:** Detailed information for each antibody used.

Antibody	RRID	Full name	Specificity	Citation	Type	Host
GluN2A	AB_2112295	NMDA Receptor 2A (GluN2A) Antibody #4205	H, M, R	(Cell Signaling Technology Cat# 4205, RRID: AB_2112295)	polyclonal	rabbit
GluN2B	AB_1264223	NMDA Receptor 2B (GluN2B) Antibody #4207	H, M, R	(Cell Signaling Technology Cat# 4207, RRID: AB_1264223)	polyclonal	rabbit
NMDAR2B Ser1480	AB_11087426	NMDAR2B (Ser1480) Antibody Rabbit pAb #bs-5382R	H, M, R	(Bioss, Cat# bs-5382R, RRID: AB_11087426)	polyclonal	rabbit
CKαII	AB_2236816	CK2α Antibody #2656	H, M, R, MK	(Cell Signaling Technology Cat# 2656, RRID: AB_2236816)	polyclonal	rabbit
Phospho-AMPA Receptor 1	AB_10860773	Phospho-AMPA Receptor 1 (GluA1) (Ser845) (D10G5) Rabbit mAb #8084	H, M, R	(Cell Signaling Technology Cat# 8084, RRID: AB_10860773)	monoclonal	rabbit
AMPA Receptor 1	AB_2732897	AMPA Receptor 1 (GluA1) (D4N9V) Rabbit mAb #13185	M, R	(Cell Signaling Technology Cat# 13185, RRID: AB_2732897)	monoclonal	rabbit
GABA(B)R1	AB_2278774	GABA(B)R1 Antibody #3835	H, M, R	(Cell Signaling Technology Cat# 3835, RRID: AB_2278774)	polyclonal	rabbit
Phospho-SAPK/JNK	AB_823588	Phospho-SAPK/JNK (Thr183/Tyr185) (81E11) Rabbit mAb #4668	H, M, R	(Cell Signaling Technology Cat# 4668, RRID: AB_823588)	monoclonal	rabbit
SAPK/JNK	AB_2250373	SAPK/JNK Antibody #9252	H, M, R, Mk, Z, B	(Cell Signaling Technology Cat# 9252, RRID: AB_2250373)	polyclonal	rabbit
GABA(B)R2	AB_2232133	GABA(B)R2 Antibody #3839	H, M, R	(Cell Signaling Technology Cat# 3839, RRID: AB_2232133)	polyclonal	rabbit
NCAM1 (CD56)	AB_2868490	NCAM1 (CD56) (E7X9M) XP® Rabbit mAb #99746	H, M, R, Mk	(Cell Signaling Technology Cat# 99746, RRID: AB_2868490)	monoclonal	rabbit
Phospho-GSK-3α/β (Ser21/9)	AB_329830	Phospho-GSK-3α/β (Ser21/9) Antibody #9331	H, M, R, Mk, Z	(Cell Signaling Technology Cat# 9331, RRID: AB_329830)	monoclonal	rabbit
GSK-3α/β	AB_10547140	GSK-3α/β (D75D3) Rabbit mAb #5676	H, M, R, Hm, Mk	(Cell Signaling Technology Cat# 5676, RRID: AB_10547140)	polyclonal	rabbit
Caspase-9	AB_2068620	Caspase-9 (C9) Mouse mAb #9508	H, M, R, Hm, Mk	(Cell Signaling Technology Cat# 9508, RRID: AB_2068620)	monoclonal	mouse
p38 MAPK	AB_10999090	p38 MAPK (D13E1) XP® Rabbit mAb #8690	H, M, R, Hm, Mk, B, Pg	(Cell Signaling Technology Cat# 8690, RRID: AB_10999090)	monoclonal	rabbit
β-actin	AB_2223172	β-Actin (13E5) Rabbit mAb #4970	H, M, R, Mk, B, Pg	(Cell Signaling Technology Cat# 4970, RRID: AB_2223172)	monoclonal	rabbit
GABA A Receptor alpha 1	AB_732498	Anti-GABA A Receptor alpha 1 antibody (ab33299)	R	(Abcam Cat# ab33299, RRID: AB_732498)	polyclonal	rabbit
PSA-NCAM IgM (12E3)	AB_2572932	PSA-NCAM Monoclonal Antibody (12E3), eBioscience™	H, M, R	(Thermo Fisher Scientific Cat# 14–9,118-82, RRID: AB_2572932)	monoclonal	mouse
Polysialic Acid-NCAM clone 2-2B, IgM	AB_95211	Anti-Polysialic Acid-NCAM Antibody, clone 2-2B	H, M, R, Vertebrates	(Millipore Cat# MAB5324, RRID: AB_95211)	2-2B, monoclonal	mouse
ST8SIA4		ST8SIA4 Rabbit Polyclonal Antibody	H, M, R	(OriGene Cat# TA370482)	polyclonal	rabbit
ST8SIA2		ST8SIA2-Specific Polyclonal antibody, Cat No. 19736–1	H, M, R	(Proteintech Cat#19736–1)	polyclonal	rabbit
Secondary AB HRP-conjugated horse anti-mouse	AB_330924	Anti-mouse IgG, HRP-linked Antibody #7076	Mouse	(Cell Signaling Technology Cat# 7076, RRID: AB_330924)	polyclonal secondary	horse
Secondary AB HRP-conjugated goat anti-rabbit	AB_2099233	Anti-rabbit IgG, HRP-linked Antibody #7074	Rabbit	(Cell Signaling Technology Cat# 7074, RRID: AB_2099233)	polyclonal secondary	goat
Secondary AB HRP-conjugated goat anti-mouse	AB_228329	Goat anti-Mouse IgM Secondary Antibody, HRP	Mouse	(Thermo Fisher Scientific Cat# 31440, RRID: AB_228329)	polyclonal secondary	goat

B, Bovine; H, Human; M, Mouse; Mk, Monkey; Pg, Pig; R, Rat; Z, Zebra; RRID, Research Resource Identification.

### 2.4 Immunoprecipitation studies

Brains were dissected, and protein lysates were prepared for hippocampus, prefrontal cortex, cortical, midbrain, and cerebellar regions as described before (Riga et al., 2014; Bhattacharya et al., 2018a). PSD-95 fractions were pulled down using Protein A/G Magnetic Beads (Millipore, Billerica, MA). Briefly, 40 μL magnetic bead slurry was washed with 500 μL of 1X IMP buffer (pH 7.4) and incubated with PSD-95 antibody for 1 h at room temperature (1:10, rabbit monoclonal antibody; Cell Signaling Technology Inc., MA). Then, 200 μg of the tissue lysate was added to the bead-antibody complex and incubated overnight at 4°C with slight agitation (Bhattacharya et al., 2017). The immunoprecipitated fraction was purified by washing it several times with 1X IMP buffer (pH 7.4). Equal amounts of samples with 5X sample-reducing buffer (Thermo Scientific, Waltham, MA) were loaded onto an SDS PAGE gel to probe for the presence of PSD-95, as well as its interaction with GluN2B receptor subunits (1:1000, rabbit monoclonal antibody; Cell Signaling Technology Inc., MA). All blots were probed with HRP-conjugated anti-rabbit secondary (1:2500, Cell Signaling Technology Inc., MA).

### 2.5 Cell culture

IMR-32 neuroblastoma cells were obtained from ATCC (CAT# CCL-127, Manassas, VA, United States) and cultured in growth medium containing Eagle’s Minimum Essential Medium (EMEM, ATCC CAT# 30-2003) supplemented with 10% fetal bovine serum (FBS). Cells were maintained at 37°C in a humidified 5% CO₂ incubator and passaged four times to ensure log-phase growth before experimentation. IMR-32 cells were selected for this study due to their well-characterized human neuroblastoma origin, their endogenous expression of PSA-NCAM and biosynthetic enzymes, ST8Sia4 and UDP-E, which are absent in most neuroblastoma cell lines. This feature makes them highly relevant for modeling Aβ-induced cytotoxicity, and PSA–NCAM–mediated interactions.

### 2.6 CRISPRa-based upregulation of ST8Sia4

A CRISPR activation (CRISPRa) system was used to upregulate transcription of the ST8Sia4 gene by targeting its promoter with designed guide RNAs. This resulted in elevated expression of ST8Sia4 and, by implication, increased PSA-NCAM levels. To assess the relationship between PSA-NCAM expression and GluN2B phosphorylation, both the engineered ST8Sia4-overexpressing IMR-32 cells and parental IMR-32 cells were co-transfected with GluN1 (SC308819, Origene Technologies Inc., Rockville, MD), GluN2B (SC119642, Origene Technologies Inc., Rockville, MD), and pMaxGFP^1^ plasmids at a 2:2:1 ratio. After 24 h, cells were harvested, lysed, and subjected to immunoblotting to quantify total GluN2B, pGluN2B (Ser1480), and CKIIα expression, with Actin (Table 1) as a loading control.

### 2.7 Aꞵ treatment

The lyophilized Amyloid-beta (human Aβ_42_) peptide was purchased from Anaspec (CAT # AS-20276, Fremont, CA, United States). Aβ_42_ peptide was dissolved in 1% NH_4_OH, followed by diluting in 1X TBS to a concentration of 1 mM. Diluted Aβ_42_ peptides were aliquoted and stored at −80°C until cells were ready for treatment. IMR32 cells were used for treatment at passages less than 11. Cells were seeded in T25 25 cm^2^ flasks (Thermo Scientific, Waltham, MA) and assessed for confluency 24 h post-plating. Cells were ready for treatment at 40–50% confluency. A 1 mM stock of Aβ_42_ was diluted in growth medium to give treatment concentrations of 1 μM, 0.5 μM, 0.25 μM, and 0.125 μM. Treatment and control flasks were harvested 24-h post-treatment for mRNA and protein analysis.

### 2.8 Protein extraction

Cell pellets were lysed on ice with extraction buffer consisting of RIPA buffer (89,900, Thermo Scientific, Waltham, MA), protease/phosphatase inhibitor cocktail (5,872, Cell Signaling Technology Inc., MA), and Triton-X-100 (1%) (52H0286, Sigma Chemical Co., St. Louis, MO) for 30 min with intermittent vortexing. The resulting lysate was centrifuged at 13,000 rpm for 5 min at 4° C, and the supernatant was collected as the cell lysate. The protein concentration in the supernatant was determined by Bradford Protein assay Reagent (Bio-Rad, Hercules, CA), using BSA protein standard (Bio-Rad, Hercules, CA).

### 2.9 Immunoblotting – cell culture

Equal amounts of protein from cell lysate (60 μg) were loaded for each sample, quantified by Bradford Assay Reagent (Bio-Rad, Hercules, CA), diluted with 5X sample-reducing buffer (Thermo Scientific, Waltham, MA), and normalized to *β*-actin as a loading control. Samples were then either stored at −80 or −20°C for later use or heated at 90°C for 5 min and resolved on 4–20% Bio-Rad Mini-PROTEAN TGX Precast Protein Gradient Gels (Bio-Rad, Hercules, CA) with Precision Plus Protein Dual Color Standard used as a ladder for molecular weight estimation (#1610374, Bio-Rad, Hercules, CA). Samples were run at 70 V for 20 min, 75 V for 20 min, and then at a constant voltage of 80 V until the bands reached the bottom of the gel. Proteins were then transferred to PVDF membranes (0.45-mM pore size, Immuno-Blot, Bio-Rad, Hercules, CA) for Western blotting for 1 h at 100 V. Membranes were blocked in TBS (20 mM Tris, 500 mM NaCl, pH 7.4) containing 1% casein (Bio-Rad, Hercules, CA) and incubated in primary antibody overnight at 4°C (Table 1). HRP-conjugated goat anti-rabbit secondary (1:2500, Cell Signaling Technology Inc., MA) and HRP-conjugated goat anti-Mouse IgM secondary (1:2500, Thermo Scientific, Waltham, MA) were incubated for 1 h at room temperature. To re-evaluate the same membrane, it was exposed to Restore Stripping Buffer (Pierce, 21,059; Thermo Scientific, Waltham, MA) for 10 min, washed with TBST, and blocked with 1% casein again before being re-blotted with the next primary antibodies. Signals from antibodies were imaged with raw films, using an Azure Biosystems C500 Imager (Azure Biosystems, Dublin, CA, United States). The blots were analyzed using Image J software (National Institutes of Health, ImageJ bundled with 64-bit Java 1.8.0_172). All immunoblotting membranes were preserved in their entirety.

### 2.10 RNA isolation and RT-qPCR

ST8Sia4, h-NCAM, ST8Sia2, UDP-E, GAPDH, and actin mRNA levels were quantified in cell pellets from Aβ treatment groups using RT-qPCR (Table 2). Following cell lysis and homogenization, total RNA was extracted using a column-based isolation method (PureLink RNA Mini Kit, Invitrogen). RNA concentrations were determined with a Tecan NanoQuant Infinite 200 Pro and standardized to 50 ng per sample. Complementary DNA (cDNA) was synthesized from the extracted RNA using the Invitrogen SuperScript III First-Strand Synthesis SuperMix with Oligo (dT) primers in a thermocycler, and subsequently diluted 1:10 with RNase-free water. For RT-qPCR, 2 μL of diluted cDNA was combined with 12.5 μL SYBR Green (Applied Biosystems PowerSYBR Green PCR Master Mix), 9.5 μL RNase-free water, and 1 μL of a forward and reverse primer mix (10 μM; Integrated DNA Technologies, Coralville, Iowa; Zhiyun et al., 2006; Al-Saraireh et al., 2013) to make a 25 μL reaction per well. Non-template controls were included to detect primer contamination and dimerization. Primer efficiency was validated using a standard curve, ensuring values ranged between 90 and 110%. Samples were loaded in triplicate onto a 96-well plate, and RT-qPCR was performed using a Roche Lightcycler 96. The cycling protocol included a 10-min preincubation at 95°C, followed by 40 cycles of amplification (95°C for 15 s, 60°C for 60 s) and a melting curve analysis (95°C for 15 s, 65°C for 15 s, with a 0.2°C/s ramp to 95°C). Cq values were collected using Roche’s Lightcycler 96 SW 1.1 software, averaged, and analyzed via the 2 − ΔΔCT method. To evaluate the effects of A*β* treatment on h-NCAM, ST8Sia4, ST8Sia2, and UDP-E gene expression, RNA from cell pellets was reverse transcribed, and RT-qPCR was conducted on the resulting cDNA, using β-actin as a control for normalization.

**Table 2:** List of primers used in this study.

Primer	Forward Sequence (5′ - 3′)	Reverse Sequence (5′ – 3′)
hNCAM	AGTCCAAGGGGAACCCAGT	CGGCTTTTCCACACAGGTTG
hNCAM (Literature)	TGC CCA TCC TCA AAT ACA AAG C	ATC AGG TTC ACT TTA ATA GAG TTT
hST8SIA2	GTGGTCTTCCTCATCTTCGCA	CAGGCTTCAGGGTTCCCTTT
hST8SIA2 (Literature)	CAG AGA TCG AAG AAG AAA TCG GG	GTG CTT ATT CTT CTT CAG TGG CG
hST8SIA4	ATCTAGCTCCTGTGGTGGAGT	GGCAGCCTGACAGTGATGAA
hST8SIA4 (Literature)	CTA CAT AGC CTC CTA CCT GAA	GGA CAC TGT CAT TCA GCA TGG
hUDP-N-acetyl-2-epimerase/N-acetylmannosamine kinase (UDP-E)	GTGCTTCGGGATGGAAACCT	TCAATGCCCTTCTTCCGCAT
hUDP-N-acetyl-2-epimerase/N-acetylmannosamine kinase (UDP-E) (Literature)	TGC CCT TCC TAT GAC AAA CTT	GCA TCA CTC GAA CCA TCT CTT
β-actin	CAT TGC TGA CAG GAT GCA GAA GG	TGC TGG AAG GTG GAC AGT GAG G
GAPDH	ACC ACA GTC CAT GCC ATC AC	TCC ACC ACC CTG TTG CTG TA

### 2.11 Statistical analysis

Statistical analyses were done using two-tailed Student’s *t*-test, and one-way analyses of variance (ANOVA) (in cases of significant effect, *post hoc* comparisons were performed using Tukey or Sidak post hoc test). Calculation of *p* values, SEM, mean, and standard deviation was performed using GraphPad Prism (v9.0.0, for Windows, San Diego, CA, United States). All significantly different data are shown with *p* < 0.05, with exact *p* values. Analyses were blinded wherever possible.

## 3 Results

### 3.1 Age-dependent decline in overall GluN2B-NMDARs and differential regulation of GABA_A_Rs characterize AD and normal brain aging

**Figure 1:**
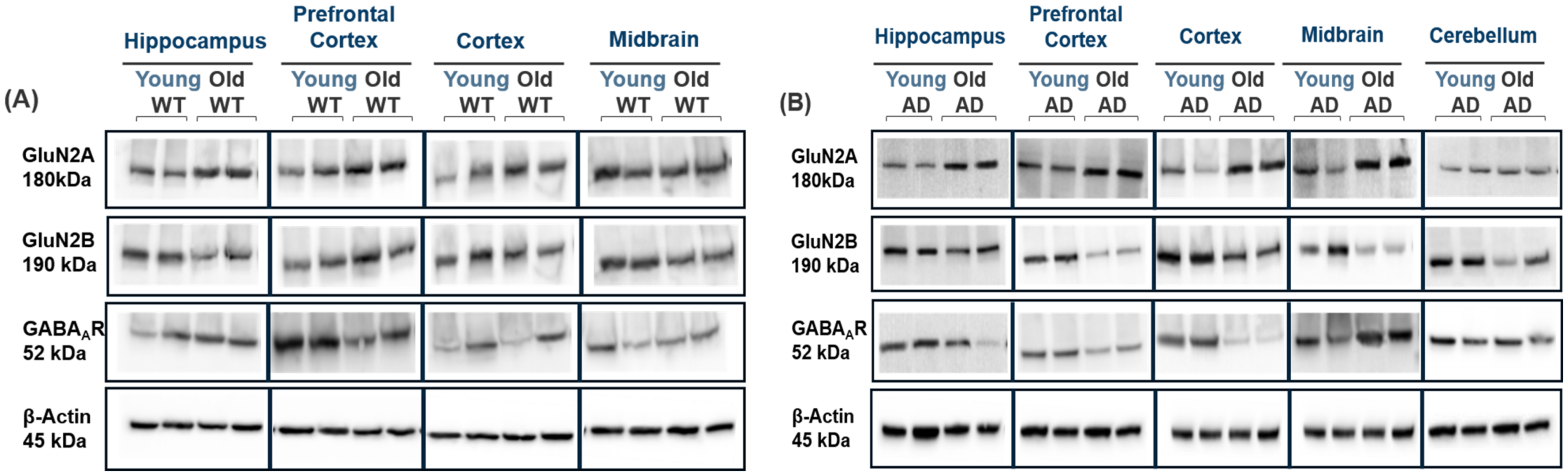
Regional expression changes in GluN2A, GluN2B, and GABA_A_R in WT and AD mice with aging. **(A)** Representative immunoblots showing GluN2A, GluN2B, and GABA_A_R expression across the hippocampus, prefrontal cortex, cortex, midbrain, and cerebellum in young and old WT mice. GluN2A expression remained largely stable with aging, except for an increase in the prefrontal cortex. GluN2B expression decreased with age across most regions, while GABA_A_R expression was reduced in the hippocampus and cortex in old WT mice. **(B)** Representative immunoblots from young and old AD mice showing GluN2A, GluN2B, and GABA_A_R levels across the same regions. GluN2A expression increased with aging in nearly all regions, while GluN2B and GABA_A_R levels decreased broadly.

**Figure 2:**
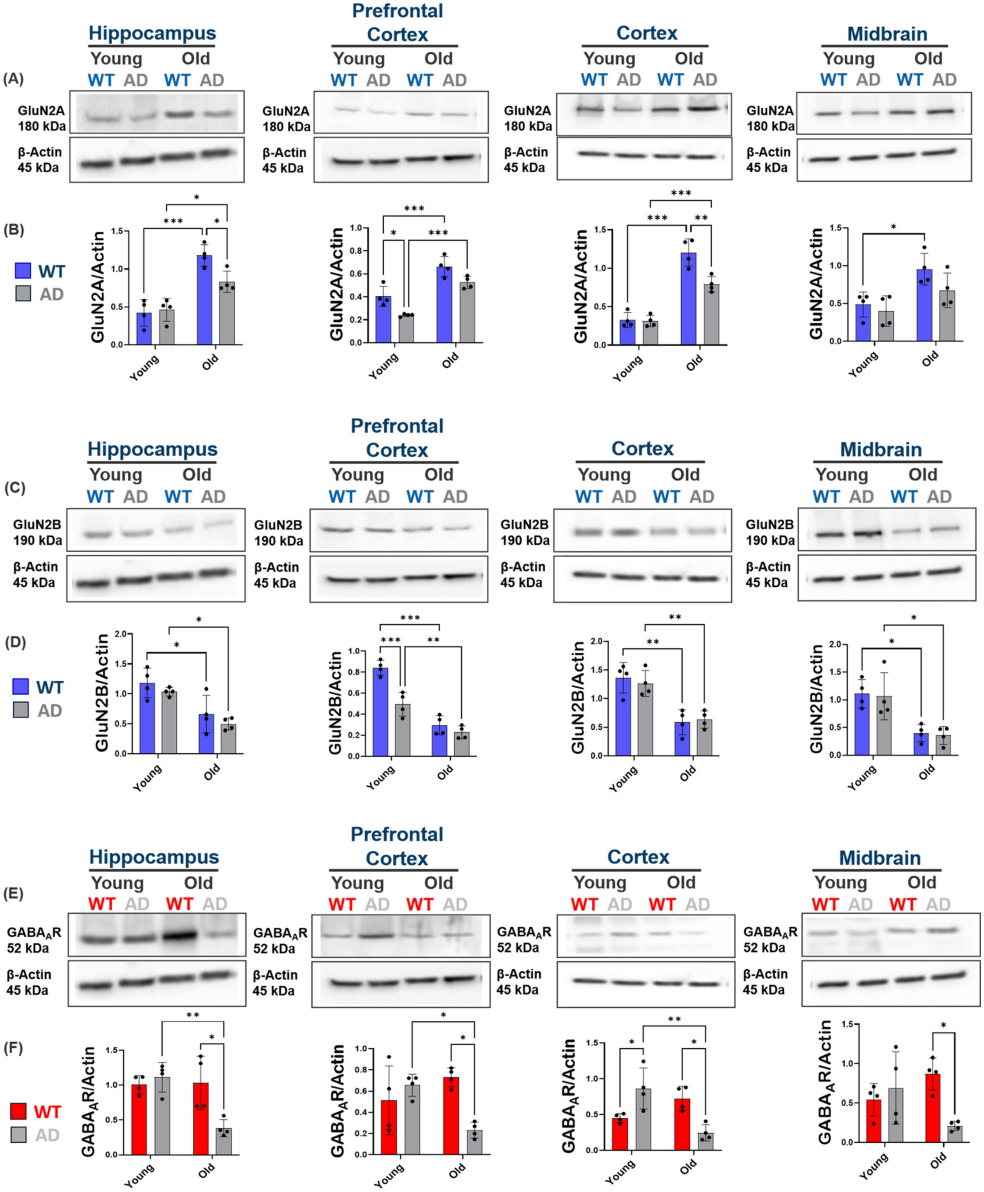
Comparative Analysis of GluN2A, GluN2B, and GABA_A_R Receptor Expression in Young and Old WT vs AD Mice. **(A,B)** Western blot analysis comparing GluN2A levels between WT and AD mice showed increased GluN2A expression in hippocampal and cortical regions of old WT mice compared to AD and a slight decrease in the prefrontal cortex of young AD mice. **(C,D)** No significant change region-wide between ages, except for an observed decrease in the prefrontal cortex of young AD mice. **(E,F)** Downregulated GABA_A_R expression is seen in old AD mice across all regions compared to WT. For each brain region, *n* = 4–5 mice per group. Bar graphs depict the mean ± SD normalized to β-actin. *p < 0.05, **p < 0.01, ***p < 0.001.

GluN2-NMDARs show distinct spatiotemporal expression patterns in the brain, with GluN2B subunit activity being predominantly prenatal, and GluN2A subunit being postnatal (Monyer et al., 1994; Laurie et al., 1997; Woodhall et al., 2001; Vieira et al., 2020). This spatiotemporal pattern is vital in the progression of several disease states (Palmer and Good, 2011; Liu et al., 2019b). We investigated the expression dynamics of GluN2A- and GluN2B-NMDAR subunits in Tg2576 and wild-type (WT) mice in the cortex, prefrontal cortex, hippocampus, and midbrain, regions associated with plasticity and implicated in AD progression, using immunoblotting (Figure color code: blue-excitatory, red-inhibitory). We analyzed WT (Figure 1A; Supplementary Figure S1), AD (Figure 1B; Supplementary Figure S2), and WT vs. AD mice in both age groups (young and old). GluN2A subunit expression showed significant increase with aging in WT and AD mice across most brain regions (hippo: ANOVA, *p* = 0.010, *F* (1, 12) = 8.18; pfc: ANOVA, *p* = 0.010, *F* (1, 12) = 8.21; ctx: ANOVA, *p* = 0.020, *F* (1, 12) = 6.01, *n* ≥ 4; Figures 2A,B), except in the midbrain which only showed significant increase in aged WT mice (mb: ANOVA, *p* = 0.030, *F* (1, 12) = 5.50, *n* ≥ 4; Figures 2A,B). In young mice, GluN2A levels were mostly unchanged between WT and AD groups, except for a slight decrease in the prefrontal cortex of AD mice (ANOVA, *p* = 0.03, *F* (1,12) = 20.4, *n* ≥ 4; Figures 2A,B). In old mice, GluN2A levels were significantly higher in WT compared to AD mice in the hippocampus (ANOVA, *p* = 0.03, *F* (1,12) = 4.92) and cortex (ANOVA, *p* = 0.007, *F* (1,12) = 9.50), with no significant differences observed in other regions (Figures 2A,2B). In contrast, GluN2B subunit expression showed significant decrease with aging in WT and AD mice region-wide (hippo: ANOVA, *p* < 0.001, *F* (1, 12) = 25.7; pfc: ANOVA, *p* < 0.001, *F* (1, 12) = 23.1; ctx: ANOVA, *p* = 0.008, *F* (1, 12) = 21.81; mb: ANOVA, *p* < 0.001, *F* (1, 12) = 27.2, *n* ≥ 4; Figures 2C,D). Additionally, GluN2B levels in young mice showed significant regional decrease in the prefrontal cortex of AD mice (ANOVA, *p* < 0.001, *F* (1,12) = 25.7, *n* ≥ 4; Figures 2C,D), while other regions remained unchanged.

In addition, we reasoned that alterations in excitatory neurotransmission may also lead to contemporary changes in inhibitory neurotransmission as a compensatory mechanism led by Gamma-aminobutyric acid receptor subtype A ionotropic channels (GABA_A_Rs). Hence, we next wanted to investigate whether these alterations in GluN2A- and GluN2B-subunit dynamics affected the excitatory-inhibitory (E/I) balance in AD. E/I Imbalance may influence NMDAR-mediated excitotoxicity in AD, via reduced expression of GABA_A_Rs, leading to decreased inhibitory signaling, contributing to overall E/I imbalance (Sheila and Sandra, 2021). Using immunoblotting, we analyzed the expression of ionotropic inhibitory GABA_A_R expression region-wide in the normal brain aging and AD aging. Immunoblotting for GABA_A_R revealed no significant changes in expression between young WT and AD mice, except for an increase in the AD mice cortices (ctx: ANOVA, *p* = 0.040, *F* (1, 12) = 24.9, *n* ≥ 4; Figures 2E,F). A significant reduction in GABA_A_R expression was observed in aged AD mice when compared to age-matched WT mice, across all regions (hippo: ANOVA, *p* = 0.010, *F* (1, 12) = 8.18; pfc: ANOVA, *p* = 0.010, *F* (1, 12) = 8.21; ctx: ANOVA, *p* = 0.020, *F* (1, 12) = 6.01; mb: ANOVA, *p* = 0.030, *F* (1, 12) = 5.50, *n* ≥ 4; Figures 2E,F). Taken together, the significant changes in the GluN2A/2B subunit expression patterns and downregulation of GABA_A_R in AD mice provide a strong indication of differential regulation and E/I imbalance in AD disease state. Analysis of other GABA-receptor subunits (B1, B2) showed a decrease in GABA_B1_R expression in WT mice with aging in prefrontal cortex and cortex, with no change in other regions (Supplementary Figure S3B). No changes in GABA_B2_R in WT mice were observed except in cortex, which showed a slight decrease in old WT mice (Supplementary Figure S3C), while in AD mice, no significant changes in GABA_B1_R across all regions were observed except in midbrain, which showed a marked increase in old AD mice (Supplementary Figure S3E). GABA_B2_R showed a significant decrease with age in AD mice across all regions except prefrontal cortex, where we observed no significant changes (Supplementary Figure S3F).

### 3.2 Extrasynaptic localization of GluN2B subunits increases with disease progression in AD

**Figure 3:**
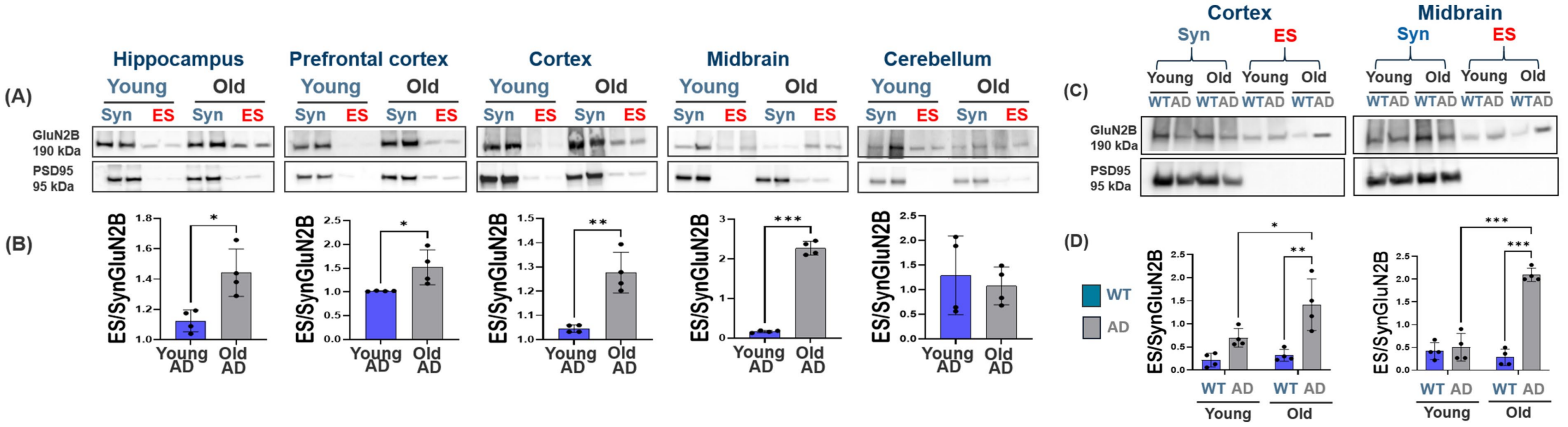
Increased extrasynaptic localization of GluN2B subunits with disease progression in AD mice and comparative analysis of ES-GluN2B and synaptic GluN2B expression in midbrain and cortex between WT and AD mice. **(A,B)** Immunoblot analysis showing significant increases in ES-GluN2B levels across brain regions of AD mice. Quantification reveals a marked increase in ES-GluN2B expression in these regions, indicating a shift from synaptic to extrasynaptic localization with disease. No significant changes in the cerebellum of AD mice. **(C,D)** Results demonstrate significant increases in ES-GluN2B levels in old AD mice compared to old WT mice, emphasizing the disease-dependent shift toward extrasynaptic localization, which becomes more pronounced with aging in these brain regions. Bar graphs represent the mean ± SD of ES-GluN2B levels normalized to Synaptic GluN2B. For each brain region, *n* = 4–5 mice per group. *p < 0.05, **p < 0.01, ***p < 0.001.

Our experiments demonstrate decreases in GluN2B subunit expression with aging in AD and WT mice. As mentioned earlier, GluN2B subunit can localize in the synapse and extrasynaptic areas, the ES-GluN2Bs being responsible for impaired plasticity (LTD). Hence, we wanted to investigate whether the decrease in GluN2B subunit expression was associated with extrasynaptic localization of GluN2B subunits (ES-GluN2B). Thus, we investigated changes in ES- versus synaptic-GluN2B subunit by analyzing pull-down of PSD-95 fragments. Immunoprecipitation (IP)-PSD-95 precipitates 95% of synaptic receptors, rendering the remaining solution containing mostly ES receptors (Xu et al., 2008; Swanger et al., 2016). Analyzing IP-PSD-95 fragments and remnants, we observed a significant increase in ES-GluN2B subunit expression levels across all brain regions with AD progression, with the most marked increase in cortex and midbrain (ctx, Student’s two-tailed t-test, *t* (4) = 5.42, *p* = 0.005; mb, Student’s two-tailed t-test, *t* (4) = 14.7, *p* < 0.001, *n* ≥ 4; Figures 3A,B), with no observed changes in the cerebellum (Student’s two-tailed *t*-test, t(4) = 0.482, *p* = 0.650, *n* ≥ 4; Figure 3B). We then characterized the ES- vs. synaptic-GluN2B subunit expression between WT and AD mice in the midbrain and cortex for both age cohorts and found significant increase in ES-GluN2B in old AD mice as compared to WT (ctx, ANOVA, *p* = 0.002, *F* (1, 12) = 12.9, *n* ≥ 4; mb, ANOVA, *p* < 0.001, *F* (1, 12) = 26.71, n ≥ 4; Figures 3C,D). These results provide additional support that increased ES-GluN2B subunit is correlated with AD progression in this model.

### 3.3 CK2α and GluN2B phosphorylation are differentially regulated in AD and normal aging

**Figure 4:**
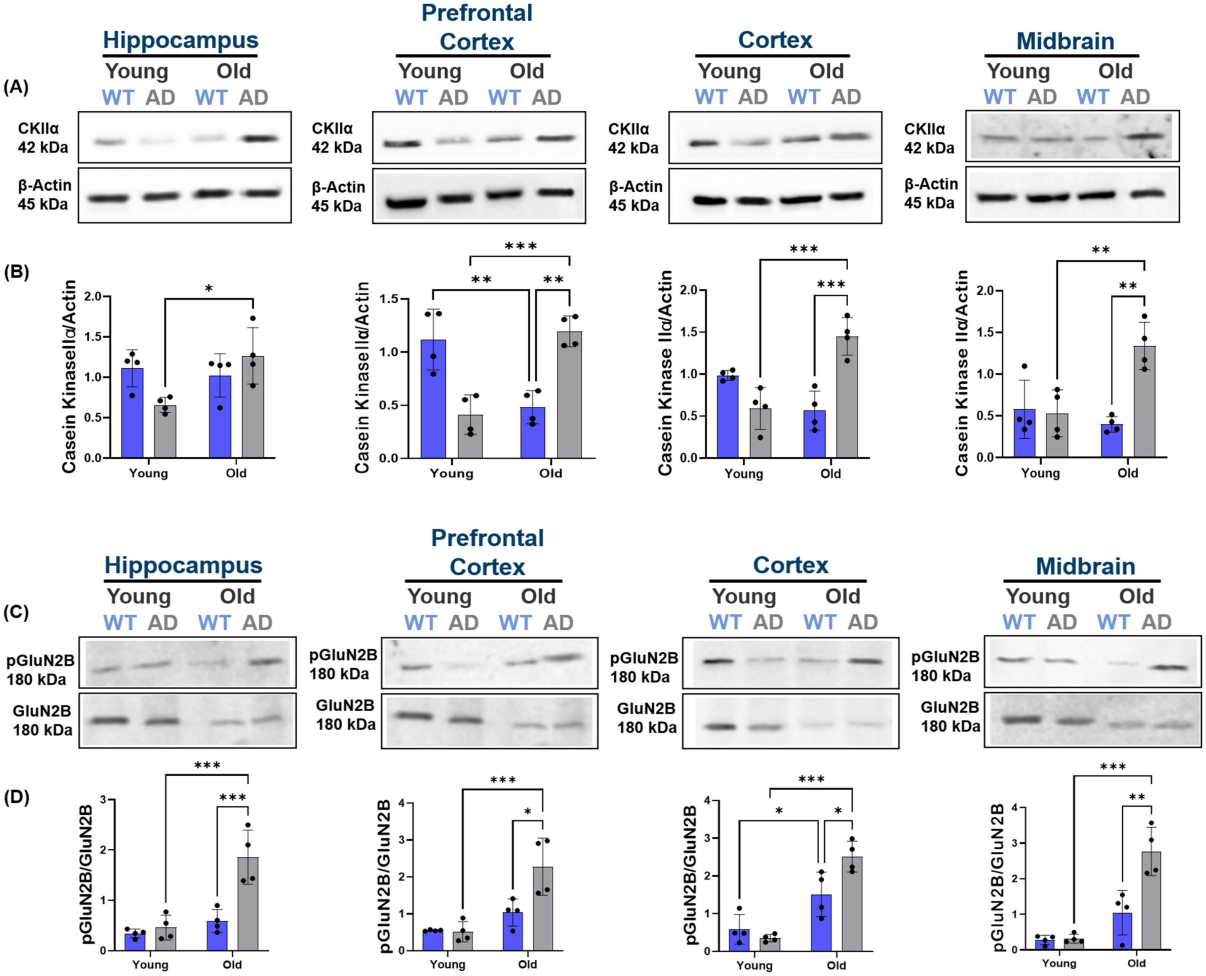
Region-specific increases in GluN2B phosphorylation and casein kinase IIα (CK2α) expression in AD brain regions. **(A,B)** Immunoblot and quantification of CK2α protein levels across brain regions (hippocampus, prefrontal cortex, cortex, and midbrain) of aged AD mice compared to aged WT mice. CK2α expression significantly increases with age in AD mice, whereas WT mice exhibit stable or reduced levels. *n* = 4–5 per group. **(C,D)** Western blot and quantification showing significant increases in phosphorylation of GluN2B at Ser1480 (pGluN2B) across brain regions (hippocampus, prefrontal cortex, cortex, and midbrain) of aged AD mice compared to aged WT mice. Old AD mice also showed higher pGluN2B levels than their WT counterparts in all regions. The ratio of pGluN2B to total GluN2B is markedly elevated in old AD mice, supporting increased extrasynaptic signaling activity with disease progression. *n* = 4–5 per group. All bar graphs depict mean ± SD of protein levels normalized to loading controls (GluN2B or β-actin). *p < 0.05, **p < 0.01, ***p < 0.001.

To understand the molecular basis of increased extrasynaptic GluN2B receptor localization in AD, we assessed the expression of casein kinase II alpha (CK2α), the enzyme known to phosphorylate GluN2B at Ser1480, thereby promoting its extrasynaptic localization, and the corresponding levels of pGluN2B. Immunoblotting revealed a significant increase in CK2α expression with age in AD mice across all brain regions examined, particularly in the prefrontal cortex and cortex (pfc, ANOVA, *p* < 0.001, *F* (1, 12) = 16.75; ctx, ANOVA, *p* < 0.001, *F* (1, 12) = 16.80, *n* ≥ 4), with no such increase observed in WT mice (Figures 4A,B). Additionally, in the pfc, young WT showed significant CK2α increase when compared to young AD (pfc, ANOVA, *p* = 0.02, *F* (1, 12) = 5.5; Figures 4A,B), with no significant changes in other regions. In contrast, CK2α expression declined slightly or remained unchanged with age in WT mice (hippo, ANOVA, *p* = 0.42 *F* (1, 12) = 0.702; pfc, ANOVA, *p* = 0.005, *F* (1, 12) = 10.79; ctx, ANOVA, *p* = 0.08, *F* (1, 12) = 3.52; midbrain, *p* = 0.93, *F* (1, 12) = 0.01, *n* ≥ 4; Figures 4A,B). These findings suggest AD-specific upregulation of this kinase may drive ES-GluN2B enrichment. To verify whether this enzymatic expression correlated with functional receptor phosphorylation, we probed for GluN2B phosphorylated at Ser1480 (pGluN2B). Quantification revealed a significant age-related increase in pGluN2B levels in AD mice across all brain regions (hippo, ANOVA, *p* < 0.001, *F* (1, 12) = 34.3; ctx, ANOVA, *p* < 0.001, *F* (1, 12) = 45.2; midbrain, ANOVA, *p* < 0.001, *F* (1, 12) = 39.7, *n* ≥ 4; Figures 4C,D), whereas pGluN2B expression remained low or decreased with aging in WT mice. Notably, in the cortex and midbrain of aged AD mice, pGluN2B levels were more than twofold higher than their WT counterparts (ctx, *p* < 0.001; mb, *p* = 0.002), consistent with the observed ES-GluN2B accumulation. Together, these results suggest that AD-associated upregulation of CK2α and subsequent Ser1480 phosphorylation of GluN2B may represent a key mechanistic driver of aberrant ES-GluN2B localization and associated excitotoxicity in aging AD brains.

### 3.4 Region-specific changes in PSA-NCAM and its biosynthetic enzymes in AD and normal brain aging

**Figure 5:**
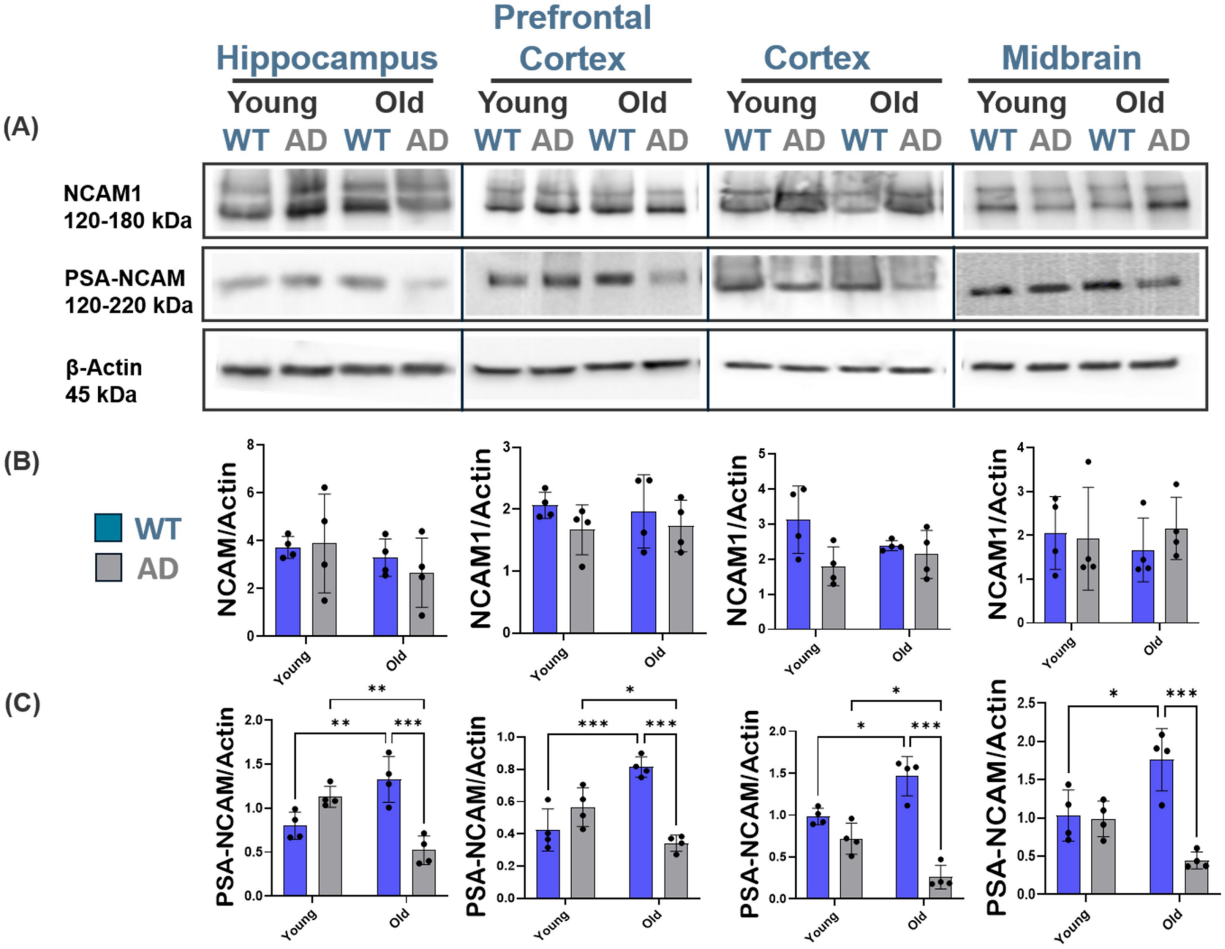
Region-specific PSA-NCAM downregulation in AD age-associated patterns. Representative Western blot images and quantification of PSA-NCAM protein expression in the cortex, prefrontal cortex, hippocampus, and midbrain of young WT and AD mice. **(A–C)** Comparison of PSA-NCAM expression between WT and AD mice reveals a significant reduction in old AD mice across all brain regions. No significant changes in NCAM expression were observed between AD and normal brain aging. For each brain region, *n* = 4–5 mice per group. Bar graphs depict the mean ± SD of NCAM and PSA-NCAM levels normalized to β-actin. *p < 0.05, **p < 0.01, ***p < 0.001.

**Figure 6:**
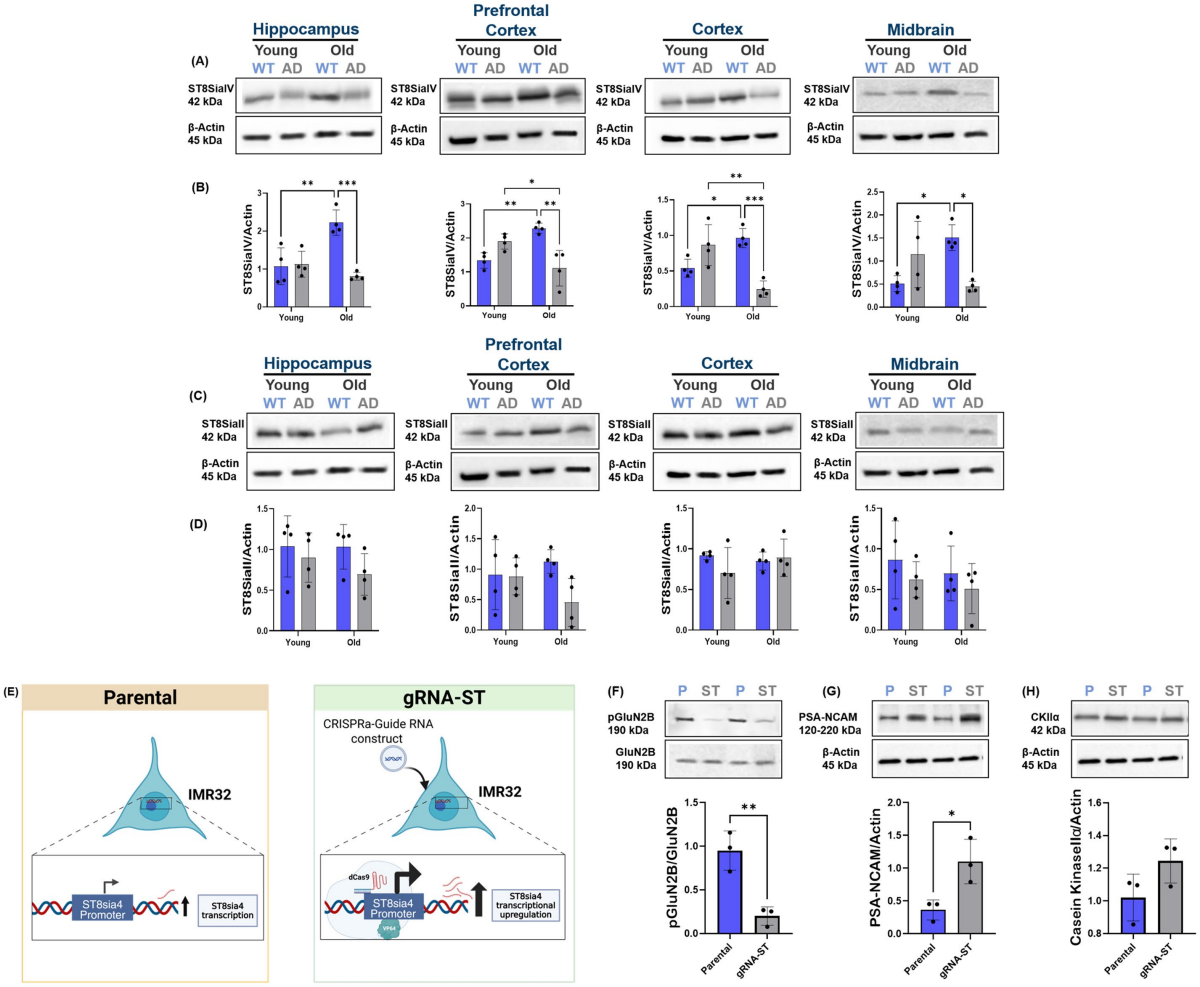
Region-specific expression of polysialylation enzymes ST8Sia2 and ST8Sia4 in AD and normal aging. **(A,B)** Western blot images and quantitative analysis of ST8Sia4 protein expression in the hippocampus, prefrontal cortex, cortex, and midbrain of young and aged WT and AD mice. ST8Sia4 expression significantly increased with age in WT mice but declined in aged AD mice across all regions, notably in the prefrontal cortex and cortex. Similarly, ST8Sia4 showed a significant decrease in aged AD mice compared to aged WT mice. **(C,D)** ST8Sia2 protein levels showed no significant regional differences. No significant changes were observed across age groups region-wide. **(E)** Parental IMR32 cells are unmodified. In gRNA-ST conditions, IMR32 cells are transfected with plasmid DNA that expresses dCas9-VP64 and the guide RNA targeting the ST8sia4 promoter, enabling CRISPR-mediated transcriptional activation of ST8sia4. **(F)** Western blot analysis revealed significantly higher GluN2B phosphorylation at Ser1480 in parental cells compared to PSA–NCAM–overexpressing cells (gRNA-ST), **(G)** increased PSA-NCAM expression in gRNA-ST cells, **(H)** with no significant difference in CKIIα levels. Bar graphs depict mean ± SD of protein levels normalized to loading controls (GluN2B or β-actin), *n* = 3 per group. *p < 0.05, **p < 0.01, ***p < 0.001.

Since our data suggest increased ES-GluN2B subunit levels with AD progression, we next wanted to investigate if increased ES-GluN2B subunit levels were due to altered PSA-NCAM expression across brain regions. PSA-NCAM is critical for both neural development and synaptic plasticity. PSA-NCAM inhibits ES-GluN2B subunit activity physiologically, and its dysregulation is associated with the loss of synaptic plasticity in AD (Varbanov and Dityatev, 2017; Giacobbo et al., 2019). Characterization of NCAM and PSA-NCAM expression in AD and WT mice with aging through immunoblotting revealed a significant reduction in PSA-NCAM expression in old AD mice when compared to age-matched WT mice, across all regions (hippo, ANOVA, *p* < 0.001, *F* (1, 12) = 36.82; pfc, ANOVA, *p* < 0.001, *F* (1, 12) = 41.5; ctx, ANOVA, *p* < 0.001, *F* (1, 12) = 72.4; mb, ANOVA, *p* < 0.001, *F* (1, 12) = 21.4, *n* ≥ 4; Figures 5A,C). However, there were no significant changes in NCAM expression between AD and WT aging (*p* < 0.05, Figure 5B). PSA-NCAM showed increased levels across all brain regions in WT aging (Supplementary Figure S4C) with a contrasting decrease in AD aging region-wide (Supplementary Figure S4F). These results indicate that deficits in PSA-NCAM may exacerbate the detrimental effects of increased ES-GluN2B and suggest its role in regulating ES-GluN2B. To further probe the cause of PSA-NCAM dysregulation, we examined expression levels of its biosynthetic enzymes, ST8Sia2 and ST8Sia4. Immunoblot analyses revealed that ST8Sia4 expression significantly decreased with age in AD mice in prefrontal and cortical regions (pfc, ANOVA, *p* = 0.030, *F* (1, 12) = 5.46; ctx, ANOVA, *p* < 0.001, *F* (1, 12) = 33.7, *n* ≥ 4), while it increased with age in WT mice (Figures 6A,B). In contrast, ST8Sia2 levels showed no consistent pattern of change between age groups or genotypes (hippo, ANOVA, *p* = 0.140, *F* (1, 12) = 2.42; pfc, ANOVA, *p* = 0.100, *F* (1, 12) = 2.95; ctx, ANOVA, *p* = 0.560, *F* (1, 12) = 0.360; mb, ANOVA, *p* = 0.240, *F* (1, 12) = 1.52, *n* ≥ 4; Figures 6C,D). These data indicate that ST8Sia4 is the dominant polysialyltransferase supporting adult PSA-NCAM expression. It is downregulated in AD aging, suggesting its contribution to the observed loss of PSA-NCAM and resulting synaptic vulnerability. Given the observed correlations between PSA-NCAM deficits and increased ES-GluN2B localization *in vivo*, we next sought to directly test this mechanistic relationship using our ST8sia4/PSA-NCAM-overexpressing CRISPRa system in IMR-32 cells (Figure 6E). Subsequently, GluN1/GluN2B and pMax-GFP plasmids were co-transfected into both parental and gRNA-ST cells. Immunoblot analyses revealed that parental IMR-32 cells exhibited significantly higher phosphorylation of GluN2B at the Ser1480 residue compared to the gRNA-ST cells (Student’s two-tailed t-test, *t* (3) = 5.04, *p* = 0.006, *n* = 3; Figure 6F), while PSANCAM expression significantly increased in gRNA-ST cells (Student’s two-tailed *t*-test, *t* (3) = 3.43, *p* = 0.03, *n* = 3; Figure 6G) with no significant difference in CKIIα levels (Student’s two-tailed *t*-test, *t* (3) = 1.17, *p* = 0.120, *n* = 3; Figure 6H).

### 3.5 Modulation of p38 MAPK/JNK apoptotic markers in AD across brain regions with AD and normal brain aging

**Figure 7:**
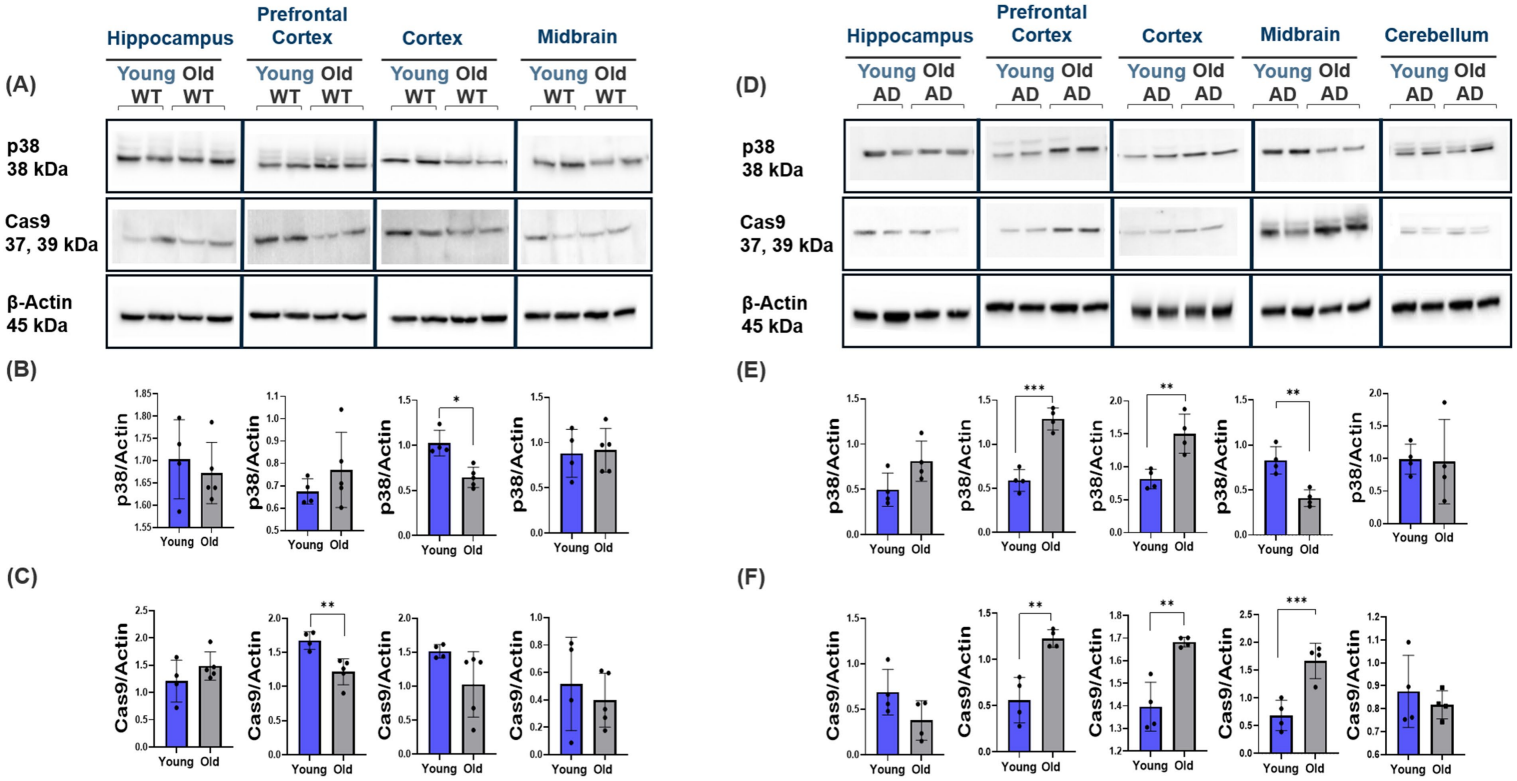
Modulation of p38 and Cas9 apoptotic markers in AD and normal brain aging. Representative Western blot images showing the expression of p38 and Cas9 in the cortex, prefrontal cortex, hippocampus, and midbrain of WT and AD mice across different ages. **(A–F)** p38 and Cas9 protein expression in the prefrontal cortex, cortex, and midbrain of AD mice increased with aging, contrasting with decreases observed in WT mice (cortex and prefrontal cortex, respectively). For each brain region, *n* = 4–5 mice per group. Bar graphs depict the mean ± SD normalized to β-actin. *p < 0.05, **p < 0.01, ***p < 0.001.

**Figure 8:**
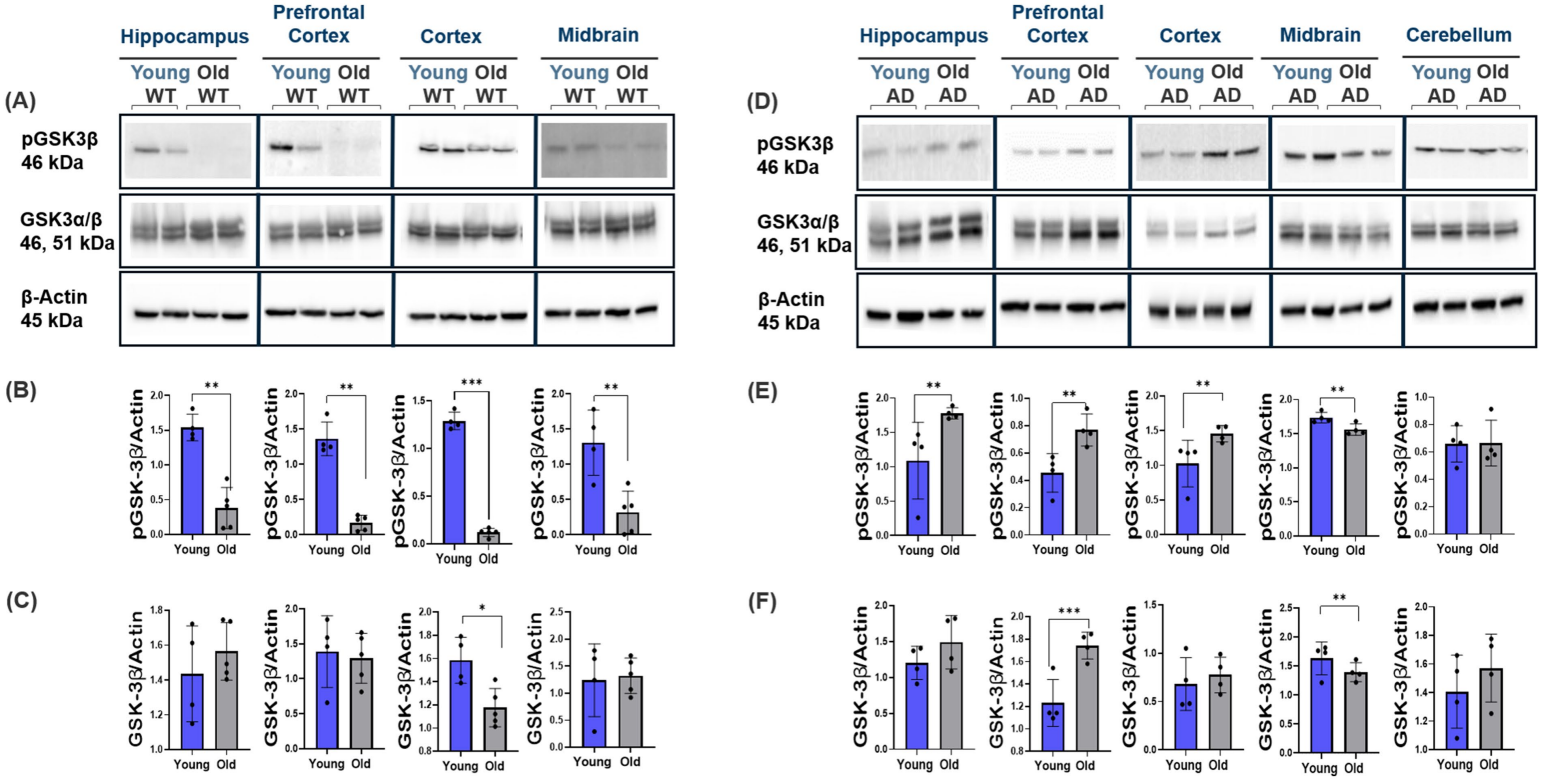
Modulation of pGSK3β and GSK3β neuroinflammatory markers in AD and normal brain aging. Representative Western blot images showing the expression of GSK3β (Total and phospho) in the cortex, prefrontal cortex, hippocampus, and midbrain of WT and AD mice across different ages. **(A–F)** Old AD mice showed significant upregulation in GSK3β phosphorylation in the hippocampus, prefrontal cortex, and cortex, whereas WT mice demonstrated downregulation across all regions with aging. For each brain region, *n* = 4–5 mice per group. Bar graphs depict the mean ± SD normalized to β-actin. *p < 0.05, **p < 0.01, ***p < 0.001.

**Figure 9:**
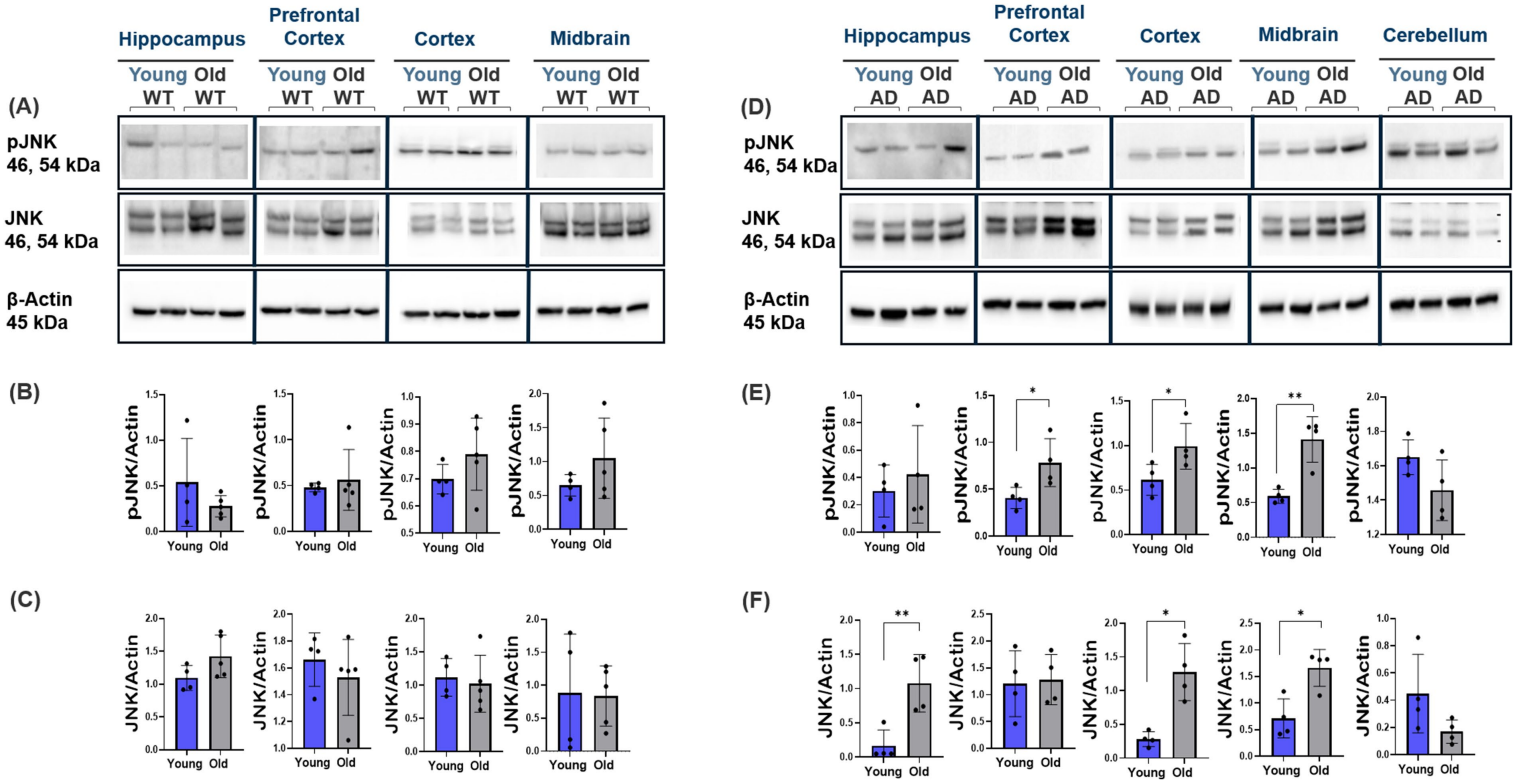
Modulation of JNK apoptotic markers in AD and normal brain aging. Representative Western blot images showing the expression of JNK (Total and phospho) in the cortex, prefrontal cortex, hippocampus, and midbrain of WT and AD mice across different ages. **(A–F)** AD mice exhibited increased JNK phosphorylation, especially in the cortex and midbrain, with no significant changes observed in WT mice across these regions. For each brain region, *n* = 4–5 mice per group. Bar graphs depict the mean ± SD normalized to β-actin. *p < 0.05, **p < 0.01, ***p < 0.001.

Results from the ES- vs. synaptic-GluN2B subunit spatiotemporal characterization show pivotal changes in ES-GluN2B subunit expression profiles. ES-GluN2Bs may mediate p38/RAS/JNK apoptotic pathway activation, promoting LTD (Hardingham et al., 2002; Li et al., 2009; Varbanov et al., 2023). We therefore studied the expression levels of key apoptotic downstream effectors in AD and normal aging. Interestingly, we observed no changes in WT mice except for the cortex and prefrontal cortex, which showed a decrease in p38 and Cas9 protein expression, respectively (Student’s two-tailed t-test, *p* < 0.001, *n* ≥ 4; Figures 7A–C) and a significant increase in p38 and Cas9 expression with aging in the prefrontal cortex, cortex, and midbrain of AD mice (Student’s two-tailed t-test, *p* < 0.010, *n* ≥ 4; Figures 7D–F). We observed a significant downregulation in p-GSK3β levels in WT mice across all regions with aging (Student’s two-tailed t-test, *p* < 0.001, *n* ≥ 4; Figures 8A–C). In contrast, we see a twofold upregulation of p-GSK3β (Ser 9) in the hippocampus, prefrontal cortex, and cortex of old AD mice, with no significant changes in the cerebellum (Student’s two-tailed t-test, *p* < 0.001, *n* ≥ 4; Figures 8D–F). Comparison of the ratio of phosphorylated to total GSK3β showed increased phosphorylation ratio across all brain regions in young WT (Supplementary Figure S5A). In AD mice, only the hippocampus and cortex showed increased phosphorylation ratio with no change in other regions (Supplementary Figure S6A). In addition, WT mice showed no changes in p-JNK levels (Thr183/Tyr185) (Student’s two-tailed t-test, *p* > 0.05, *n* ≥ 4; Figures 9A–C), while in AD mice, the prefrontal cortex, cortex, and midbrain showed about twofold upregulation in p-JNK (Student’s two-tailed t-test, *p* < 0.001, *n* ≥ 4; Figures 9D–F). The ratio of phosphorylated to total JNK showed no changes in WT mice (Supplementary Figure S5B) and AD mice region-wide, except prefrontal cortex, which increased significantly with aging (Supplementary Figure S6B).

### 3.6 Aβ-treatment induced modulation of PSA-NCAM and ST8Sia4 expression in IMR-32 neuroblastoma cells

**Figure 10:**
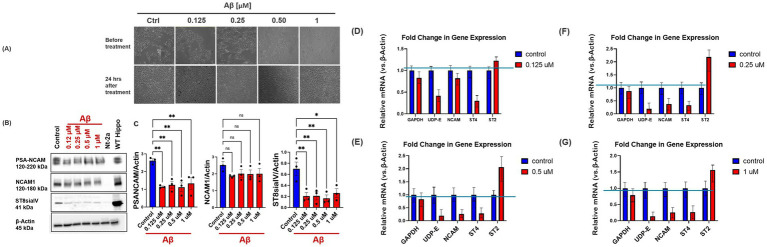
Effect of Aβ treatment on NCAM, PSA-NCAM, UDP-N-acetylglucosamine-2-epimerase, and Polysialyltransferases (ST8Sia2 and 4) Expression Levels in IMR32 Cells. **(A–C)** Western blot analysis of NCAM, PSA-NCAM, and ST8Sia4 in IMR32 neuroblastoma cells treated with increasing concentrations of Aβ (0.125–1 μM) shows a dose-dependent decrease in PSA-NCAM and ST8Sia4 protein expression. **(D–G)** Quantitative RT-PCR analysis revealed trends consistent with immunoblotting. ST8Sia4 and UDP-E expression levels were significantly downregulated, with ST8Sia4 reduced by approximately 66% and UDP-E to below half of control levels. Alternatively, ST8Sia2 mRNA expression increased across all treatment groups. β-actin was used as a loading control for normalization in all panels. Bar graphs depict the mean ± SD vs. control. *n* = 3 per treatment group. *p < 0.05; **p < 0.01; ***p < 0.001.

**Figure 11:**
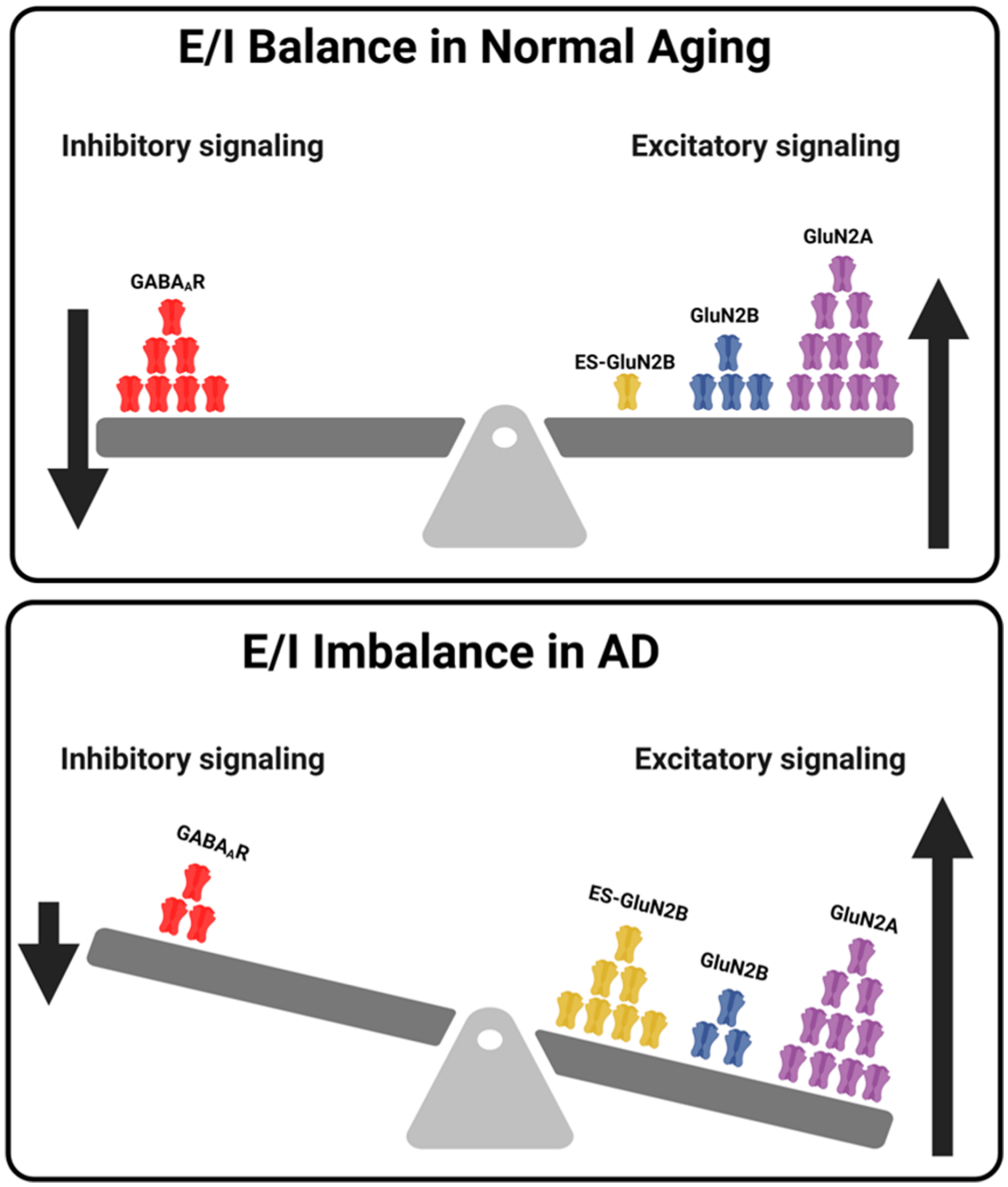
Summary of NMDA-GABA receptor-mediated E/I Balance in Normal Aging and AD. The figure illustrates the shift in E/I balance during normal aging (top) and Alzheimer’s disease (bottom). In normal aging, the balance is maintained by decreased total GluN2B level and increasing GluN2A subunit expression. In contrast, AD shows a significant decrease in GluN2B expression and an upregulation of GluN2A, alongside a marked reduction in GABA_A_R expression, reflecting diminished inhibitory signaling and overall E/I imbalance. Additionally, increased ES-GluN2B localization further contributes to synaptic dysfunction and excitotoxicity in AD.

Secretion of Aβ monomers is central to AD pathology, and the impact of Aβ on NCAM, PSA-NCAM, and sialyltransferase enzymes is not fully understood, yet these molecules play critical roles in neural plasticity, cell signaling, and cognitive functions (Schaeffer et al., 2011; Rendeiro et al., 2014; Varbanov and Dityatev, 2017). Understanding how Aβ affects these molecules at both the protein and mRNA levels could provide significant insights into aspects of the molecular mechanisms underlying AD pathology. To evaluate whether ST8Sia2 and ST8Sia4, PSA-NCAM, and UDP-E are affected by the pathological hallmark of AD, Aβ, we analyzed the protein and mRNA expression levels of NCAM, PSA-NCAM, ST8Sia4, ST8Sia2, and UDP-E in our cell model expressing the ST8 enzymes and PSA-NCAM simultaneously (IMR 32; ATCC, Manassas, VA). We treated the cells with varying concentrations of Aβ (1 μM, 0.5 μM, 0.25 μM, and 0.125 μM), with a no-treatment control (Figure 10A). Analysis of the protein expression PSA-NCAM and ST8Sia4 revealed a significant decrease (ANOVA; ST8Sia4, *p* = 0.006, *F* (4, 10) = 7.07, *n* = 3; PSA-NCAM, *p* = 0.003, *F* (4, 10) = 8.45, *n* = 3; Figures 10B,C) across all treatment concentrations in comparison to vehicle, with no significant decrease in NCAM expression (ANOVA; NCAM, *p* = 0.36, *F* (4, 10) = 1.24, *n* = 3; Figure 10C). We also analyzed the mRNA expression levels of NCAM, PSA-NCAM, ST8Sia4, ST8Sia2, and UDP-E in our cell model (IMR-32). In line with the results obtained from immunoblotting analysis, quantitative RT-PCR revealed similar trends. Notably, ST8Sia4, ST8Sia2, and UDP-E mRNA levels demonstrated biologically significant changes, meeting both statistical (*p* < 0.05) and fold-change (log₂FC > 1 or <−1) thresholds. ST8Sia4 expression decreased by approximately 66% (*p* < 0.05), while UDP-E expression dropped to <0.5-fold of vehicle levels (*p* < 0.05). ST8Sia2 expression increased by ~180% across treatment groups (*p* < 0.01; Figures 10D–G). In contrast, although NCAM mRNA levels showed a statistically significant reduction (*p* < 0.05), the fold change did not exceed the threshold for biological significance. Together, these results demonstrate that Aβ-induced neurochemical changes promote modulation of PSA-NCAM and its biosynthetic enzymes at the transcriptional and post-transcriptional levels (Figure 11).

## 4 Discussion

This study provides novel insights into the spatiotemporal regulation of ES-GluN2B and PSA-NCAM in AD mouse models and normal aging, highlighting key molecular alterations associated with synaptic dysfunction and cognitive decline in AD. Our findings demonstrate AD-specific increases in ES-GluN2B expression and a significant downregulation in PSA-NCAM levels, suggesting that Aβ-induced downregulation of biosynthetic enzymes ST8Sia4 and UDP-E contributes to a reduction in PSA-NCAM. These changes are accompanied by disruptions in excitatory/inhibitory (E/I) balance and enhanced pro-apoptotic signaling pathways, underscoring their critical role in AD pathophysiology. Using AD mice (Westerman et al., 2002; Lilja et al., 2013) and *in-vitro* models, we investigated the spatiotemporal regulation of ES-GluN2B NMDARs and PSA-NCAM in normal aging and AD-related aging across brain regions associated with neural plasticity and implicated in AD. The present study also showed mechanistic modulation of neuronal adhesion proteins by Aβ-induced neurochemical alterations, indicating the molecules or pathways as potential therapeutic targets in AD. Our data reveal a marked decrease in GluN2B expression in the prefrontal cortex of young AD mice, suggesting early synaptic vulnerability in AD. When examining age-related changes, GluN2A expression significantly increases with age in AD mice across most brain regions, while WT mice show a more modest, region-specific increase, limited to the prefrontal cortex. This suggests that GluN2A upregulation may represent a compensatory response to synaptic stress in AD (Parsons and Raymond, 2014). However, when comparing old WT and old AD mice, GluN2A levels are significantly higher in WT mice in both the hippocampus and cortex, indicating that despite AD-related aging-associated increases, GluN2A expression remains lower than in age-matched WT. This highlights regional vulnerabilities and impaired compensatory plasticity in AD mice brains (Wisniewski and Sigurdsson, 2010). Furthermore, GABA_A_R expression significantly declines in aging AD mice across all regions, while remaining stable in WT aging. This selective loss of inhibitory signaling contributes to E/I imbalance and excitotoxicity, key features of AD pathophysiology (Marín, 2012; Xu et al., 2020) (Figure 11).

We also highlight the differential localization of GluN2B subunits in synaptic versus extrasynaptic regions, crucial for understanding neurodegeneration and synaptic plasticity in AD. The balance between synaptic and extrasynaptic NMDAR activity is essential for maintaining synaptic health (Barria and Malinow, 2005; Snyder et al., 2005). With AD progression, we observed a significant shift toward increased ES-GluN2B subunit localization, particularly in the cortex and midbrain. This shift is significant because ES-GluN2B activation is linked to LTD, synaptic weakening, excitotoxicity, and neurodegeneration (Massey et al., 2004; Papouin et al., 2012). The mechanisms driving this increased ES-GluN2B localization likely involve lateral diffusion, Aβ pathology, and neuroinflammation (Snyder et al., 2005; Chiu C. Q. et al., 2019). Aβ oligomers are known to disrupt synaptic NMDAR function, promoting the relocation of GluN2B to extrasynaptic sites, which enhances Ca^2+^ influx through ES-NMDARs, triggering pathways leading to synaptic loss and neuronal death (Hardingham et al., 2002; Leveille et al., 2008). Importantly, we observed a significant increase in GluN2B phosphorylation at Ser1480 in AD mice brains, which coincided with elevated expression of CK2α, the primary kinase responsible for this site-specific modification. Phosphorylation at Ser1480 promotes the detachment of GluN2B from synaptic scaffolding proteins like PSD-95 and facilitates its lateral diffusion into extrasynaptic compartments, thereby reinforcing the neurotoxic ES-GluN2B signaling axis (Sanz-Clemente et al., 2010; Chiu C. Q. et al., 2019). Our findings show that this increase in ES-GluN2B is particularly pronounced in the cortical regions of AD mice, critical for cognitive function and affected early in the disease process (Li et al., 2016). The significant rise in ES-GluN2B levels in these regions of aged AD mice compared to WT suggests that they are especially vulnerable to the pathological effects of AD-related changes.

Our region-specific analysis revealed a striking divergence in PSA-NCAM expression between aging WT and AD mice. In WT animals, PSA-NCAM levels increased with age, particularly in the hippocampus and cortex, suggesting a role in adaptive synaptic remodeling. In contrast, AD mice showed a significant age-related decline in PSA-NCAM, pointing to disrupted synaptic plasticity and disease progression (Bisaz et al., 2011; Seki and Rutishauser, 1998; Varbanov and Dityatev, 2017; Giacobbo et al., 2019; Gascon et al., 2010). While previous studies have reported general declines in PSA-NCAM with age (Bonfanti, 2006), our data differentiate between physiological aging and AD pathology. To understand this divergence, we examined the expression of the polysialyltransferases ST8Sia2 and ST8Sia4. ST8Sia4, the dominant enzyme maintaining PSA-NCAM in the adult brain, was significantly reduced in aging AD mice, but not in WT animals. ST8Sia2 levels remained unchanged across groups, suggesting a limited role in postnatal regulation (Highet et al., 2023). These findings link the loss of PSA-NCAM in AD to decreased ST8Sia4 expression and reduced polysialylation. This enzymatic decline may stem from impaired neuronal activity-dependent regulation. Prior studies show that PSA-NCAM expression responds dynamically to neural activity (Bonfanti, 2006; Seki, 2002). In normal aging, increased synaptic activity may drive PSA-NCAM upregulation, while in AD, chronic excitotoxicity, Aβ burden, and neuroinflammation likely accelerate its degradation or downregulate its synthesis.

The interplay between increased ES-GluN2B and decreased PSA-NCAM in AD is particularly noteworthy. ES-GluN2B is pro-LTD, leading to synaptic loss and dysfunction (Massey et al., 2004; Kochlamazashvili et al., 2010; Papouin et al., 2012), and in contrast, PSA-NCAM inhibits ES-GluN2B, promoting synaptic plasticity and LTP, which are essential for normal brain function (Senkov et al., 2006; Dityatev et al., 2004). Our findings suggest that the reduction in PSA-NCAM with age in AD mice may exacerbate the detrimental effects of increased ES-GluN2B, thereby accelerating synaptic dysfunction and cognitive decline. Moreover, the selective decline in PSA-NCAM in hippocampal and cortical regions underscores the vulnerability of these areas to AD-related synaptic deficits and highlights the potential of PSA-NCAM as a therapeutic target, as seen in other studies (Senkov et al., 2006; Varbanov and Dityatev, 2017; Pinky et al., 2023; Varbanov et al., 2023). A limitation of our study is that we analyzed brain region homogenates and not cell-type–specific or laminar changes. Future studies using layer-specific microscopy will be necessary to localize PSA-NCAM and ES-GluN2B expression with greater cellular precision. To mechanistically validate this inverse relationship between PSA-NCAM expression and ES-GluN2B migration, we employed a CRISPRa-based transcriptional activation system in IMR-32 cells to selectively upregulate ST8Sia4, thereby increasing PSA-NCAM expression. Following co-transfection with GluN1/GluN2B plasmids, we observed a significant reduction in phosphorylation of GluN2B at the Ser1480 residue in gRNA-ST cells compared to parental IMR-32 cells. pGluN2B is known to mediate receptor migration to extrasynaptic sites, a process regulated by CKIIα (Sanz-Clemente et al., 2013; Chung et al., 2004; Chiu A. M. et al., 2019). The attenuation of Ser1480 phosphorylation in PSA-NCAM-enriched cells, despite unchanged CKIIα levels, suggests that PSA-NCAM can regulate GluN2B retention at the synapse. Together, these data support a novel mechanistic pathway through which PSA-NCAM suppresses the ES-GluN2B shift and promotes synaptic anchoring, thereby preserving plasticity and mitigating excitotoxic risk in the context of AD.

Activity of ST8Sia4 and UDP-E is essential for PSA-NCAM synthesis, a key modulator of synaptic plasticity and remodeling (Bonfanti, 2006; Hildebrandt et al., 2007). In the context of AD, where synaptic deficits are prominent, understanding how these enzymes are affected by AD pathological hallmarks, such as Aβ, is essential. For the first time, our results demonstrate a significant decrease in PSA-NCAM and ST8Sia4 protein expression following Aβ treatment, establishing a mechanistic link between Aβ-induced reductions in PSA-NCAM and impaired ST8Sia4 activity in AD. Additionally, we observed concordant downregulation of ST8Sia4 and UDP-E at the mRNA level, suggesting that Aβ disrupts polysialylation not only post-translationally but also at the level of gene expression. This coordinated disruption likely compromises synaptic integrity and contributes to cognitive decline (Sato and Hane, 2018; Mikkonen et al., 1999; Varbanov et al., 2023). Interestingly, ST8Sia2 mRNA levels increased, possibly as a compensatory response (Brusés and Rutishauser, 1998), although this was insufficient to restore PSA-NCAM levels. These findings underscore a dominant role for ST8Sia4 and reveal a novel Aβ-driven mechanism that impairs neural plasticity, potentially converging with elevated ES-GluN2B to promote synaptic dysfunction in AD (Li et al., 2011; Rönicke et al., 2011). Future experiments to delineate the molecular mechanisms underlying Aβ’s and tau’s effects on polysialyltransferase regulation and PSA-NCAM expression may offer novel therapeutic opportunities.

In addition to these findings, our analysis of JNK, p38, Caspase-9, and GSK3β highlights key signaling differences between AD and normal aging. In AD mice, widespread upregulation of phosphorylated JNK (Thr183/Tyr185) and p38 MAPK suggests activation of neuroinflammatory and apoptotic pathways linked to tau hyperphosphorylation and Aβ accumulation (Borsello and Forloni, 2007; Coulthard et al., 2009; Asih et al., 2020; Di Benedetto et al., 2022). In contrast, WT mice showed no significant pJNK changes, reinforcing the divergence from pathological aging. Elevated pGSK3β (Ser9), an inhibitory marker, appeared earlier in WT mice and increased only in aged AD mice, indicating dysregulated tau-related signaling in AD (Hernández et al., 2013). Region-specific increases in p38 and caspase-9 in AD brains, particularly the cortex and midbrain, point to enhanced apoptotic signaling (Li et al., 2011; Yuan and Yankner, 2000; Bredesen, 2009). Meanwhile, their decline in WT cortices may reflect a protective adaptation absent in AD (Tamayev et al., 2012; Martínez et al., 2011; Suzen et al., 2022). Summarily, our results show that Aβ treatment leads to a significant decrease in PSA-NCAM, ST8Sia4, and UDP-E expression, linking Aβ to impaired polysialylation and synaptic dysfunction in AD. Additionally, the downregulation of GluN2B and GABA_A_R, and the upregulation of ES-GluN2B via increased pGluN2B in AD mice indicate disruption of synaptic balance and a shift toward neurotoxic pathways. We demonstrate that enhancing PSA-NCAM expression and targeting ES-GluN2B may offer therapeutic strategies to promote synaptic anchoring of GluN2B, mitigate synaptic dysfunction, and rescue cognitive decline in AD. Additionally, our study provides a valuable region-specific spatiotemporal profile of receptor and polysialylation pathway alterations across multiple brain areas. This novel foundational insight sets the stage for future work aimed at delineating the precise interaction between PSA-NCAM and GluN2B, along with cellular and layer-specific regulatory mechanisms contributing to AD pathology.

## Data Availability

The original contributions presented in the study are included in the article/Supplementary material, further inquiries can be directed to the corresponding author/s.

## Footnotes

^1^Addgene plasmid #177825; https://www.addgene.org/177825/; RRID: Addgene_177,825.
