# Supplementary material for: Spatiotemporal differential regulation of extrasynaptic GluN2B receptor subunits and PSA-NCAM in brain aging and Alzheimer’s disease
Source: Front Neurosci. 2025 Aug 29;19:1649625. doi: 10.3389/fnins.2025.1649625 (PMC12426952; doi:10.3389/fnins.2025.1649625)
Supplement: Supplementary file 2 [file Table_1.DOCX]

**(A)**

**Figure S1: Expression changes in NMDA and GABA-A receptors with normal aging in WT mice.**

(A) Quantitative analysis of Western blots showing expression levels of GluN2A, GluN2B, and GABA-A receptor subunits in the hippocampus, prefrontal cortex, cortex, midbrain, and cerebellum of young and old WT mice. GluN2A expression was largely unchanged across regions, except for the prefrontal cortex, which showed a significant increase with aging (p = 0.01). In contrast, GluN2B expression significantly decreased with age across all regions except the cerebellum (p = 0.07). GABA-A receptor expression decreased significantly in the hippocampus and cortex of old WT mice, with no significant changes observed in other regions. For each brain region, n = 4–5 mice per group. Bar graphs represent mean ± SEM, normalized to β-actin. ns = not significant, *p < 0.05, **p < 0.01, ***p < 0.001.

**(A)**

**Figure S2: Expression changes in NMDA and GABA-A receptors with aging in AD mice.**

(A) Quantitative analysis of Western blots showing expression levels of GluN2A, GluN2B, and GABA-A receptor subunits in the hippocampus, prefrontal cortex, cortex, midbrain, and cerebellum of young and old AD mice. A significant increase in GluN2A expression was observed with aging in all regions (*p < 0.05 to ***p < 0.001), except in the cerebellum, where the change was not significant. GluN2B expression significantly decreased with age in all brain regions, though the cerebellum showed only a marginal (non-significant) decrease. GABA-A receptor expression decreased significantly across most regions in old AD mice, including the hippocampus, prefrontal cortex, cortex, and midbrain. For each brain region, n = 4–5 mice per group. Bar graphs represent mean ± SEM, normalized to β-actin. ns = not significant, *p < 0.05, **p < 0.01, ***p < 0.001.


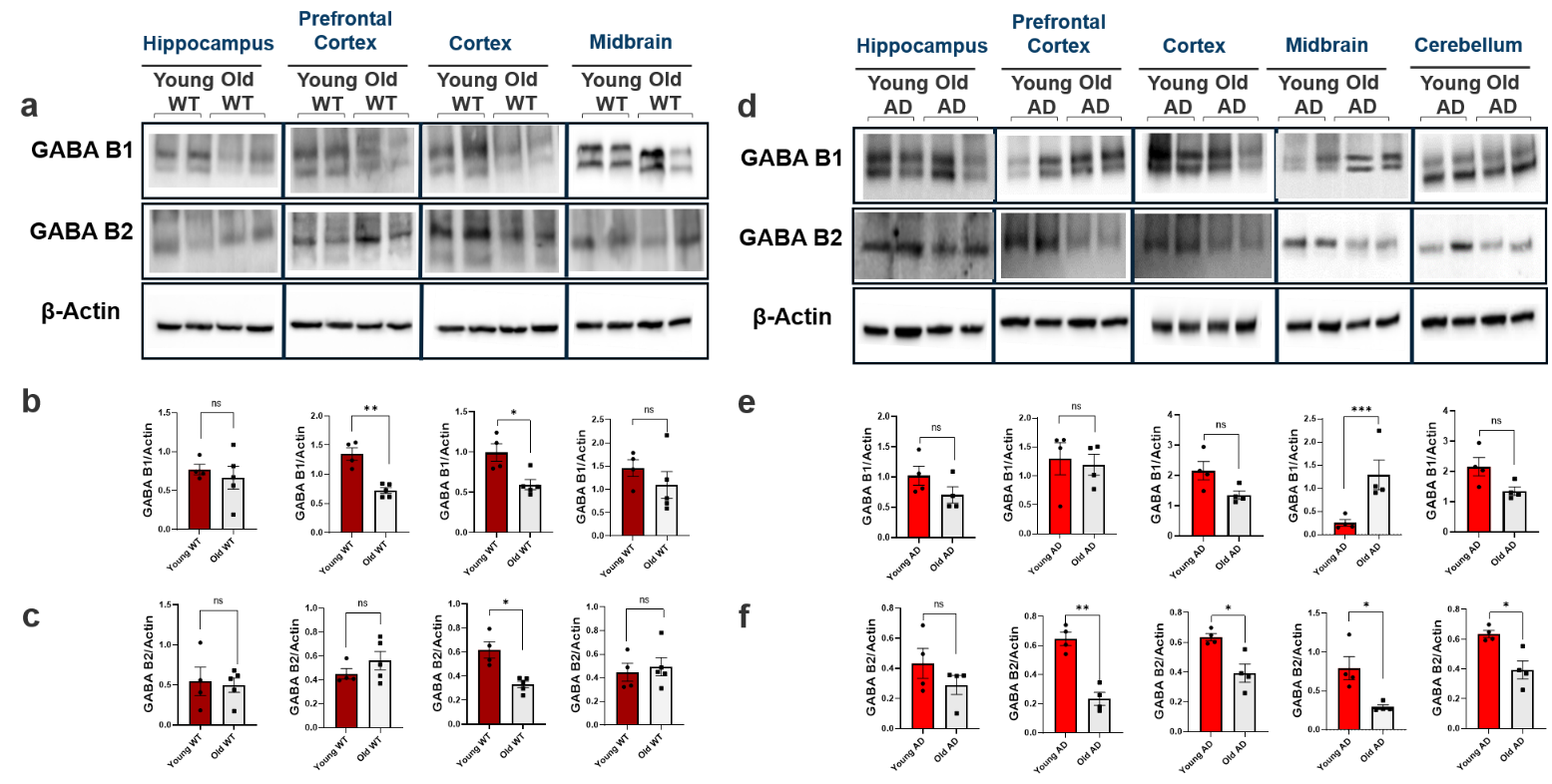


**Figure S3: Expression changes in GABA (B1, B2) receptors in normal aging and AD.** (A & D) Representative Western blot images showing the expression of GABA B1 and GABA B2 in the cortex, prefrontal cortex, hippocampus, midbrain, and cerebellum of WT and AD mice across different ages. (B) Quantitative analysis of western blots indicates a decrease in GABA B1 expression in WT mice with aging in the prefrontal cortex and cortex. Other regions show no significant change. (C) No significant changes were observed in GABA B2 expression in WT mice across all regions except in the cortex, which showed a slight significant decrease in old WT mice. (E) In AD mice, no significant changes were observed in GABA B1 expression across all regions except in the midbrain, which showed a marked increase in old AD mice. (F) GABA B2 showed a significant decrease with age in AD mice across all regions except the prefrontal cortex, where we observed no significant changes. For each brain region, *n* = 4-5 mice per group. Bar graphs depict the mean ± SEM normalized to β-actin. ns-not significant, *p < 0.05, **p < 0.01, ***p < 0.001.


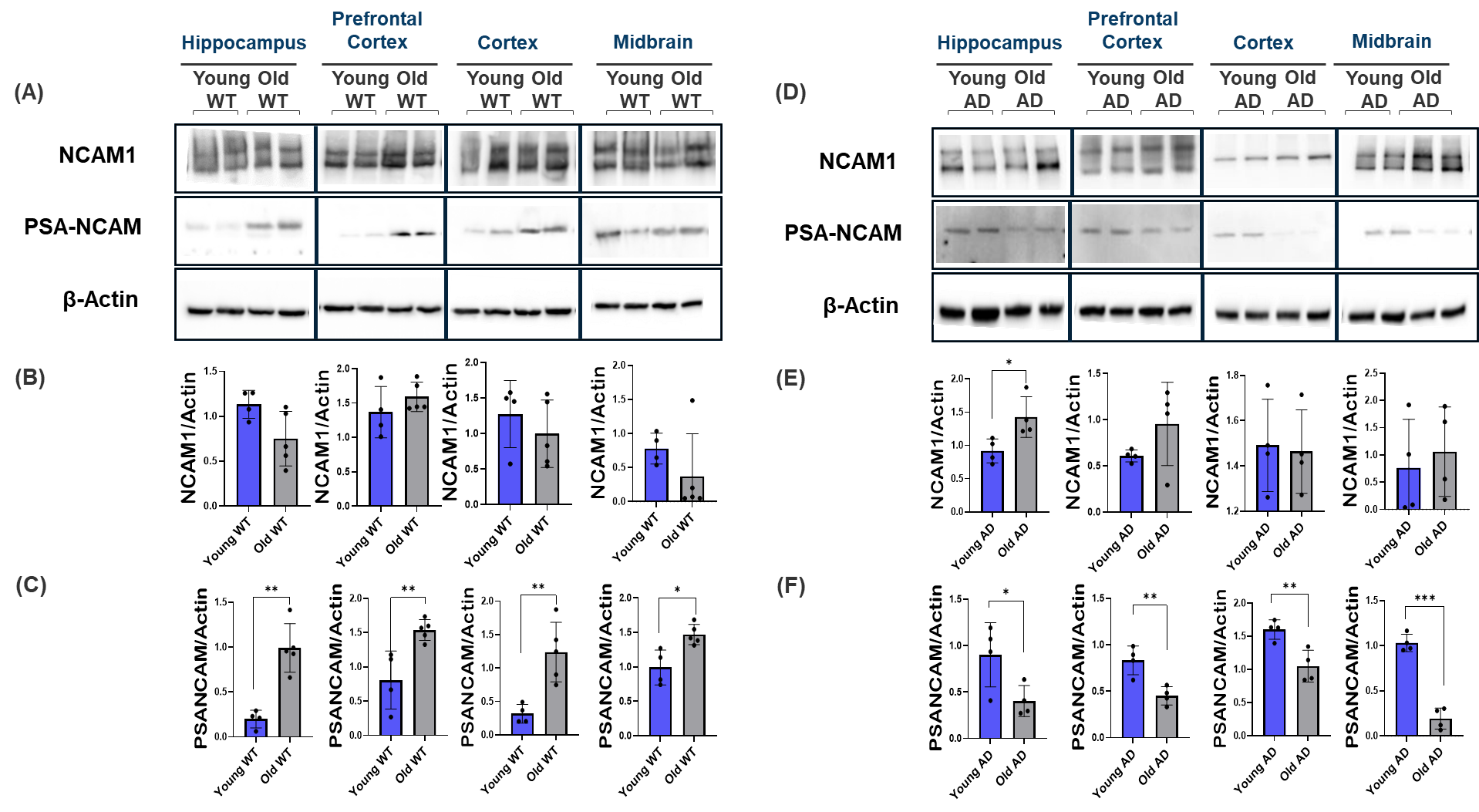


**Figure S3: Regional expression changes in NCAM and PSA-NCAM with aging in WT and AD mice.**

(A–C) Representative western blots and quantification of NCAM1 and PSA-NCAM expression in the hippocampus, prefrontal cortex, cortex, and midbrain of WT mice across age groups. PSA-NCAM expression significantly increased with aging in WT mice across all regions, particularly in the hippocampus and cortex (p < 0.001 and p = 0.006, respectively). NCAM1 levels remained largely unchanged with age. (D–F) Corresponding western blots and quantification in AD mice. In contrast to WT, PSA-NCAM expression significantly decreased with age across all regions in AD mice (*p < 0.05 to **p < 0.001). NCAM1 levels remained unchanged except in the hippocampus, where a modest increase was observed with aging (p = 0.03). n = 4–5 mice per group. Data are normalized to β-actin and represented as mean ± SEM. ns = not significant, *p < 0.05, **p < 0.01, ***p < 0.001.


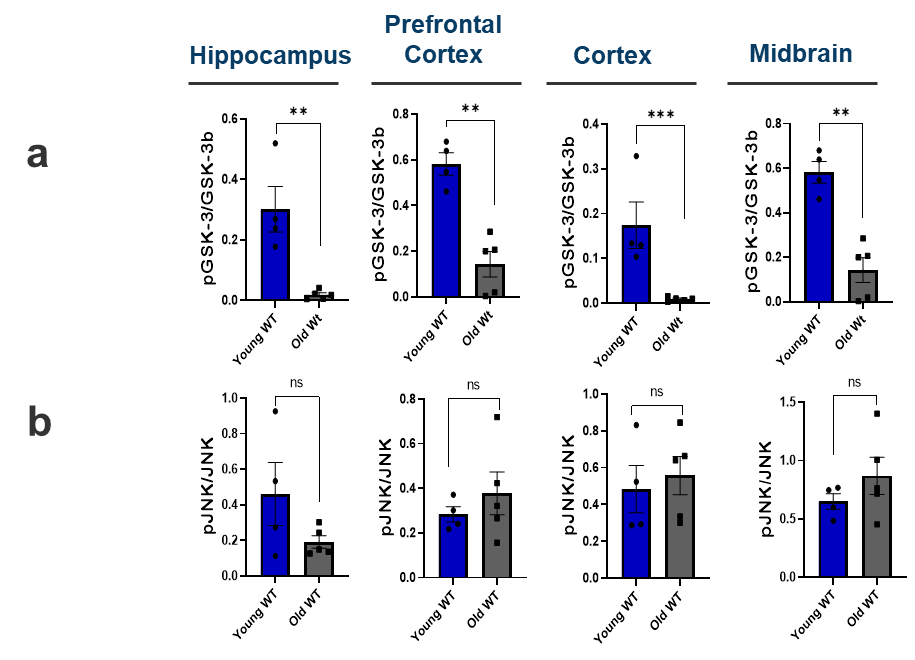


**Figure S5: Phosphorylation changes in GSK3β and JNK apoptotic markers in normal brain aging.** (A) Comparison of the ratio of phospho to total GSK3β expression in the cortex, prefrontal cortex, hippocampus, and midbrain of young and old WT mice. The young WT showed increased phosphorylation ratio across all brain regions. (B) Comparison of the ratio of phospho to total JNK expression in the cortex, prefrontal cortex, hippocampus, and midbrain of young and old WT mice. No significant changes were observed in WT mice across these regions for both age groups. For each brain region, *n* = 4-5 mice per group. Bar graphs depict the mean ± SEM normalized to β-actin. ns-not significant, *p < 0.05, **p < 0.01, ***p < 0.001.


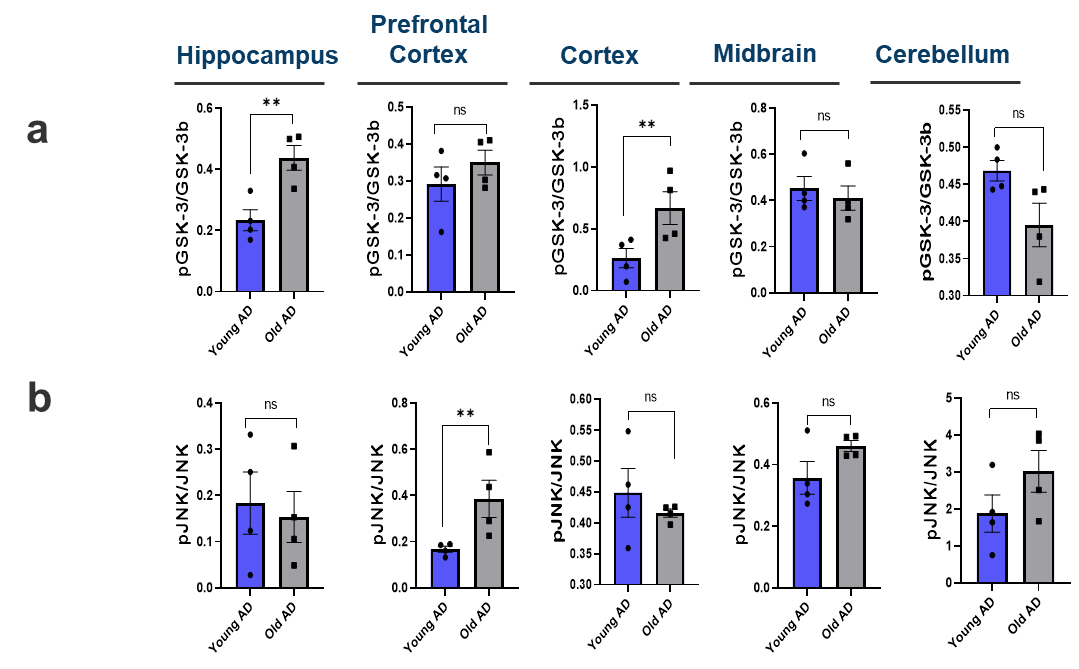


**Figure S6: Phosphorylation changes in GSK3β and JNK apoptotic markers in AD.** (A) Comparison of the ratio of phospho to total GSK3β expression in the cortex, prefrontal cortex, hippocampus, and midbrain of young and old AD mice. (B) Comparison of the ratio of phospho to total JNK expression in the cortex, prefrontal cortex, hippocampus, and midbrain of young and old AD mice. For each brain region, *n* = 4-5 mice per group. Bar graphs depict the mean ± SEM normalized to β-actin. ns-not significant, *p < 0.05, **p < 0.01, ***p < 0.001.


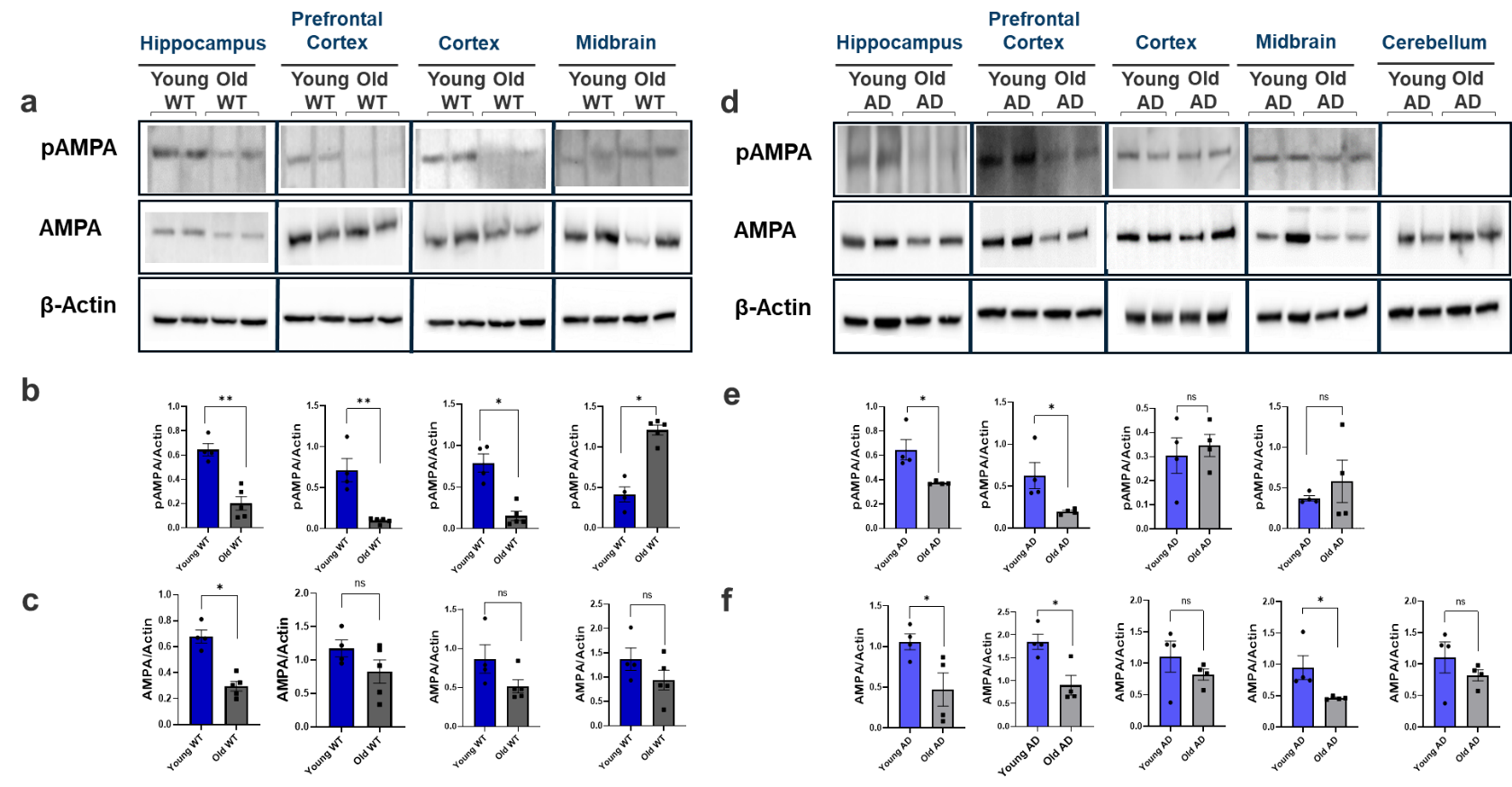


**Figure S7: Phosphorylation and expression changes in AMPA receptor in normal aging and AD.** (A & D) Representative Western blot images showing the expression of pAMPA and AMPA in the cortex, prefrontal cortex, hippocampus, and midbrain and cerebellum of WT and AD mice across different ages. (B) Quantitative analysis of western blots indicates a decrease in AMPA phosphorylation across all brain regions in WT mice with aging except midbrain which shows pAMPA expression increase with normal aging. (C) No significant changes were observed in total AMPA expression in WT mice across all regions except in hippocampus which showed a slight significant decrease in old WT mice. (E) In AD mice, hippocampal and prefrontal cortices showed significant decrease in AMPA phosphorylation with aging, while the cortex and midbrain showed no significant changes. (F) Similar trend was observed as total AMPA expression reduced significantly in hippocampus, prefrontal cortex and midbrain of old AD mice with no significant changes in other brain regions. For each brain region, *n* = 4-5 mice per group. Bar graphs depict the mean ± SEM normalized to β-actin. ns-not significant, *p < 0.05, **p < 0.01, ***p < 0.001.


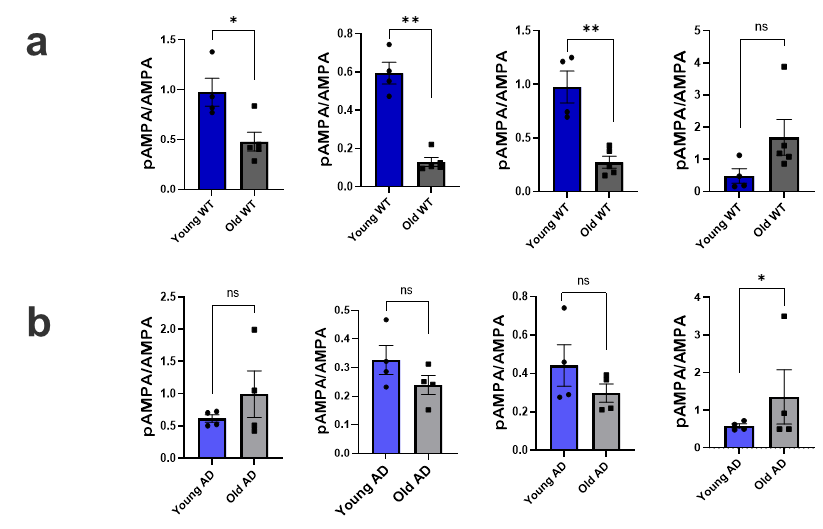


**Figure S8: Phosphorylation changes in AMPA receptor in normal aging and AD.** (A) Comparison of ratio of phospho to total AMPA expression in the cortex, prefrontal cortex, hippocampus, and midbrain of young and old WT mice. (B) Comparison of ratio of phospho to total AMPA expression in the cortex, prefrontal cortex, hippocampus, and midbrain of young and old AD mice. For each brain region, *n* = 4-5 mice per group. Bar graphs depict the mean ± SEM normalized to β-actin. ns-not significant, *p < 0.05, **p < 0.01, ***p < 0.001.
